# Sampling Points-Independent Identification of the Fractional Maxwell Model of Viscoelastic Materials Based on Stress Relaxation Experiment Data

**DOI:** 10.3390/ma17071527

**Published:** 2024-03-27

**Authors:** Anna Stankiewicz

**Affiliations:** Department of Technology Fundamentals, Faculty of Production Engineering, University of Life Sciences in Lublin, 20-612 Lublin, Poland; anna.m.stankiewicz@gmail.com

**Keywords:** viscoelasticity, linear relaxation modulus, fractional Maxwell model, stress relaxation test, experiment randomization, differentiable Lipchitz models

## Abstract

Considerable development has been observed in the area of applying fractional-order rheological models to describe the viscoelastic properties of miscellaneous materials in the last few decades together with the increasingly stronger adoption of fractional calculus. The fractional Maxwell model is the best-known non-integer-order rheological model. A weighted least-square approximation problem of the relaxation modulus by the fractional Maxwell model is considered when only the time measurements of the relaxation modulus corrupted by additive noises are accessible for identification. This study was dedicated to the determination of the model, optimal in the sense of the integral square weighted model quality index, which does not depend on the particular sampling points applied in the stress relaxation experiment. It is proved that even when the real description of the material relaxation modulus is entirely unknown, the optimal fractional Maxwell model parameters can be recovered from the relaxation modulus measurements recorded for sampling time points selected randomly according to respective randomization. The identified model is a strongly consistent estimate of the desired optimal model. The exponential convergence rate is demonstrated both by the stochastic convergence analysis and by the numerical studies. A simple scheme for the optimal model identification is given. Numerical studies are presented for the materials described by the short relaxation times of the unimodal Gauss-like relaxation spectrum and the long relaxation times of the Baumgaertel, Schausberger and Winter spectrum. These studies have shown that the appropriate randomization introduced in the selection of sampling points guarantees that the sequence of the optimal fractional Maxwell model parameters asymptotically converge to parameters independent of these sampling points. The robustness of the identified model to the measurement disturbances was demonstrated by analytical analysis and numerical studies.

## 1. Introduction

For several decades, fractional-order rheological models have been used to describe, analyze and improve the viscoelastic properties of different materials. In addition to theoretical research dedicated to fractional-order rheological models [1,2,3,4,5], hundreds of studies have been conducted on the applicability of such models for specific materials to describe their mechanical properties. The applicability of such models to the description of different polymers is well known, for example, poly-isobutylene [4], polyurea and PET [6], shape memory polymers [7], amorphous polymers [8] and flax fiber-reinforced polymer [9]. Fractional viscoelastic models are also used for modeling laminated glass beams in the pre-crack state under explosive loads [10]; stress relaxation behavior of glassy polymers [11]; description of fiber-reinforced rubber concrete [12]; viscoelastic modeling of modified asphalt mastics [13]; and modeling rate-dependent nonlinear behaviors of rubber polymers [14]. The modeling and simulation of viscoelastic foods, for example, food gums [15], carrot root [16], fish burger baking [17], is another field of application of rheological fractional models. Due to the non-integer order of the operations of integration and differentiation, the fractional-order models have improved flexibility and better adjustment to material characteristics, both in the time and frequency domains, compared to those of the classic integer-order models.

Although over the last several decades different fractional differential models have been proposed for modeling the viscoelastic processes in materials, the fractional Maxwell model (FMM) is the best known [4,5]. The relaxation modulus of the FMM, described by the product of Mittag-Leffler and inverse power functions, allows for the modeling of a very wide range of stress relaxation processes in materials. Describing the rheological properties of polymers by the FMM [18,19] is well known. However, the FMM was also applied, for example, for computational modeling and analysis on the damping and vibrational behaviors of viscoelastic composite structures [20], viscoelastic flow in a circular pipe [21], effect of temperature on the dynamic properties of mixed surfactant adsorbed layers at the water/hexane interface [22,23] and constitutive equations of the Mn-Cu damping alloy [24]. Fractional viscoelasticity described by the Maxwell model turned out to model both exponential and non-exponential relaxation phenomena in real materials.

Different identification methods for the recovery of the parameters of the non-integral-order models, including the FMM, from both static [16,25,26,27,28] and dynamic [12,29,30,31] experiments data have been proposed so far. It is known that different identification methods in association with different experiment plans result in different identification data yield models, which may differ [32]. Generally, the identification result, i.e., the chosen model, is influenced by the three entries that are necessary for model identification: the set of models from which the best model is chosen, the rule for the optimal model selection and the measurement data obtained in the experiment [32,33]. For the selected class of models, here, the set of the fractional Maxwell models, the identified model depends on the identification rule and the experiment data. The model parameters are usually determined by guaranteeing the “best-possible” fit to the measurements. Therefore, parameters of the optimal model are dependent on the measure applied for evaluating the “best” [32]. The mean-square approximation error is the predominant selection of the model quality measure, which results in a standard least-squares identification task. For the selected identification index, the model identified is usually dependent, sometimes even very strongly, on the experiment data. This is the case with FMM identification methods known in the literature [12,16,25,26,27,28,29,30,31]. This paper deals with the problem of the FMM identification using measurement data from the stress relaxation test. Therefore, the sampling instants used in the experiment and discrete-time measurements of the relaxation modulus compose the set of the experiment data. To build the optimal fractional Maxwell model whose parameters do not depend on sampling instants applied in the stress relaxation test is the aim of this paper.

In the previous paper [33], the problem of the least-squares approximation of the relaxation modulus has been considered for an assumed wide class of relaxation modulus models. Models being continuous, differentiable and Lipschitz continuous with respect to the parameters have been assumed. The main results in [33] refer to the models that are determined asymptotically, when the number of measurements tend to infinity. Whenever some applicability conditions concerning the chosen class of models are satisfied, the asymptotically optimal FMM parameters can be determined using the measurement data obtained for sampling instants selected randomly due to the appropriate randomization, even when the true relaxation modulus description is completely unknown. For the exponential Maxwell and the exponential stretched Kohlrausch–Williams–Watts models, the applicability conditions are satisfied [33]. It should be noted that the concept of identification being measurement point-independent comes from the Ljung paper [34] and the paper of [35], in which the optimal identification problems for dynamic and static systems have been considered.

In this paper, the concept of introducing an appropriate randomization for the selection of sampling instants at which the measurements of the relaxation modulus are recorded is applied for the fractional Maxwell model identification. Following [33], the real material description is completely unknown and only the measurement data of the relaxation modulus are available for model identification. Identification consists of determining the FMM that solves the problem of an optimal least-squares approximation of a real relaxation modulus. The complicated form of the relaxation modulus of the FMM (the product of Mittag-Leffler and inverse power functions) implies that the applicability of the sampling points-independent identification for FMM identification is not obvious. It is known that the relaxation modulus of the FMM is continuous and differentiable with respect to its four parameters [36]. However, the satisfaction of the Lipschitz continuous property with the bounded Lipschitz constant is proved in this paper for the first time, to guarantee the applicability of the experiment randomization concept.

A complete identification scheme leading to the strongly consistent estimate of the optimal model was specified. Assuming that the measurements are corrupted by additive disturbances, the stochastic-type analysis of the model convergence was carried out, and the exponential rate of convergence was demonstrated both analytically and by numerical studies. For materials described by the unimodal Gauss-like spectrum of relaxation used to describe the rheological properties of the materials [37,38,39] and by the Baumgaertel, Schausberger and Winter (BSW) spectrum [40,41] successfully applied for modeling the polymers [42,43], based on the simulation experiments, both the asymptotic properties and noise robustness of the algorithm were numerically studied. To improve the clarity of this article, the proof of the new FMM Lipschitz property is moved to Appendix A. The tables with the results of the numerical studies are given in Appendix B.

## 2. Materials and Methods

### 2.1. Material

A linear viscoelastic material subjected to small deformations for which the uniaxial, non-aging and isotropic stress–strain equation is given by a Boltzmann superposition equation [44]

(1) $$\varsigma \left(t\right)=\int_{-\infty }^{t}G\left(t-\tau \right)\frac{d\varepsilon \left(\tau \right)}{d\tau }d\tau$$

is considered, where $\varsigma \left(t\right)$ and $\varepsilon \left(t\right)$ are, respectively, the stress and strain and $G\left(t\right)$ denotes the linear relaxation modulus. By Equation (1), the stress $\varsigma \left(t\right)$ at time t depends on the earlier history of the strain rate described by the first-order derivative $\frac{d\varepsilon \left(\tau \right)}{d\tau }$ via the kernel given by the relaxation modulus $G\left(t\right)$.

The modulus $G\left(t\right)$ is the stress induced in the material described by constitutive Equation (1) by the unit step strain $\varepsilon \left(t\right)$ imposed. It is assumed for the studied material that the mathematical description of the modulus $G\left(t\right)$ is completely unknown. However, the real relaxation modulus $G\left(t\right)$ is accessible by measurement with a certain accuracy for an arbitrary time t∈T. Here, $T=\left[t_{0},T\right]$ with the initial time $t_{0}>0$ and T≤∞.

We make the following assumption [33]:

**Assumption** **1.** *The relaxation modulus* $G\left(t\right)$ *of the material is bounded on* T*, i.e.,* $\underset{t\in T}{\sup}G\left(t\right)\leq M<\infty$.

### 2.2. Fractional Maxwell Model

Constitutive equation of the fractional order Maxwell model is as follows [2,4,45]:

(2) $$\tau_{r}^{\alpha -\beta }\frac{d^{\alpha -\beta }\varsigma \left(t\right)}{dt^{\alpha -\beta }}+\varsigma \left(t\right)=G_{e}\tau_{r}^{\alpha }\frac{d^{\alpha }\varepsilon \left(t\right)}{dt^{\alpha }},$$

where $G_{e}$ denotes the elastic modulus, $\tau_{r}$ means the relaxation time, α and β are non-integer positive orders of fractional derivatives of the strain $\varepsilon \left(t\right)$ and stress $\varsigma \left(t\right)$, respectively. In this paper, $\frac{d^{\alpha }}{dt^{\alpha }}f(x)=D_{t}^{\alpha }f(x)$ means the fractional derivative operator in the sense of Caputo’s of a function f(x) of non-integer-order α with respect to variable t and with a starting point at t=0, which is defined by [1,4]

$$D_{t}^{\alpha }f\left(t\right)=\frac{1}{\Gamma \left(n-\alpha \right)}\int_{0}^{t}{\left(t-1\right)}^{n-\alpha -1}\frac{d^{n}}{dt^{n}}f\left(t\right)dt,$$

where n−1<α<n and $\Gamma \left(n\right)$ is Euler’s gamma function [1] (Equation (A.1.1)).

**Figure 1:**
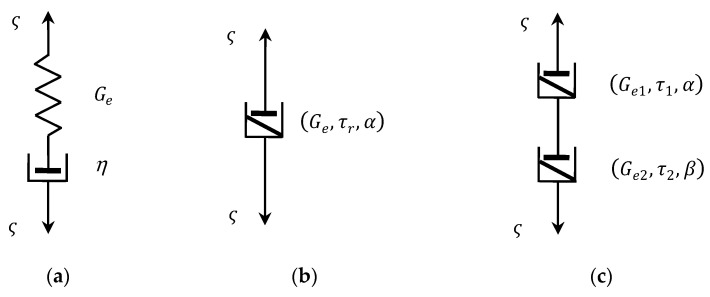
Viscoelastic models: (**a**) classic Maxwell model; (**b**) fractional Scott-Blair model of an order α; (**c**) fractional Maxwell model; elastic modulus $G_{e}$, $G_{e1}$, $G_{e2}$, viscosity η, relaxation times $\tau_{r}$, $\tau_{1}$, $\tau_{2}$.

The FMM (2) can be considered as a generalization of the classic viscoelastic Maxwell model being the series connection of the ideal spring with a dashpot (see Figure 1a) described by a differential equation of the first order [44,46]:

(3) $$\frac{d\varsigma \left(t\right)}{dt}+\frac{1}{\tau_{r}}\varsigma \left(t\right)=G_{e}\frac{d\varepsilon \left(t\right)}{dt},$$

with the elastic modulus $G_{e}$ of the spring, the relaxation time $\tau_{r}=\eta /G_{e}$, where η means the viscosity of the dashpot.

A series connection (see Figure 1c), analogical to the classic Maxwell model, of two elementary fractional Scott-Blair elements $\left(G_{e1},\tau_{r1},\alpha \right)$ and $\left(G_{e2},\tau_{r2},\beta \right)$, both described by the fractional differential equation of the general form [2,4,45]

(4) $$\varsigma \left(t\right)=G_{e}\tau_{r}^{\alpha }\frac{d^{\alpha }\varepsilon \left(t\right)}{dt^{\alpha }},$$

with the parameters $\left(G_{e},\tau_{r},\alpha \right)$ (see Figure 1b), yields the FMM described by Equation (2), where the parameters $\left(G_{e1},\tau_{r1},\alpha \right)$ and $\left(G_{e2},\tau_{r2},\beta \right)$ uniquely determine the parameters E and $\tau_{r}$ of the FMM (2); for details, see [16]. The four parameters $\left(G_{e},\tau_{r},\alpha ,\beta \right)$ of the FMM (2), compared with only two parameters $\left(G_{e},\eta \right)$, or equivalently $\left(G_{e},\tau_{r}\right)$ of the classic Maxwell model (3), are important for the improvement in the FMM accuracy and flexibility.

The uniaxial stress response of the FMM (2) imposed by the unit step strain $\varepsilon \left(t\right)$, i.e., the time-dependent relaxation modulus $G\left(t\right)$, for an arbitrary 0<β<α≤1 is given by the formula [2,4,5]:

(5) $$G\left(t\right)=G_{e}{\left(\frac{t}{\tau_{r}}\right)}^{-\beta }E_{\alpha -\beta ,1-\beta }\left(-{\left(\frac{t}{\tau_{r}}\right)}^{\alpha -\beta }\right),$$

where $E_{\kappa ,\mu }\left(x\right)$ is the generalized two-parameter Mittag-Leffler function defined by series being convergent in the whole z-complex plane [1,2]:

(6) $$E_{\kappa ,\mu }\left(x\right)=\sum_{n=0}^{\infty }\frac{x^{n}}{\Gamma \left(\kappa n+\mu \right)}.$$

Further, for the description of the FMM identification task, relaxation modulus model (5) is denoted as

(7) $$G_{M}\left(t,g\right)=G_{e}{\left(\frac{t}{\tau_{r}}\right)}^{-\beta }E_{\alpha -\beta ,1-\beta }\left(-{\left(\frac{t}{\tau_{r}}\right)}^{\alpha -\beta }\right),$$

to emphasize the dependence on a four-element vector of model parameters

(8) $$g={\left[\begin{array}{cccc} \alpha & \beta & G_{e} & \tau_{r} \end{array}\right]}^{T},$$

where the subscript ‘M’ means the model.

For the special case α=β, the FMM (2) reduces to the Scott-Blair model (compare (4))

(9) $$2\varsigma \left(t\right)=G_{e}\tau_{r}^{\alpha }\frac{d^{\alpha }\varepsilon \left(t\right)}{dt^{\alpha }},$$

and the relaxation modulus is described by

(10) $$G_{M}\left(t,g\right)=\frac{G_{e}}{2\Gamma \left(1-\alpha \right)}{\left(\frac{t}{\tau_{r}}\right)}^{-\alpha }.$$

Let us consider the following set of the FMM admissible model parameters:

(11) $$G=\left\{g:\beta_{0}\leq \;\beta \leq \alpha \leq 1;\;G_{emin}\leq G_{e}\leq G_{emax};\tau_{rmin}\leq \tau_{r}\leq \tau_{rmax}\right\}$$

where $\beta_{0}>0$ is an arbitrarily small positive number and the maximal and minimal values of elastic modulus $G_{e}$ and relaxation time $\tau_{r}$ follow from the a priori knowledge concerning the material under investigation and are such that $G_{emin}>0$ and $\tau_{rmin}>t_{0}$. G is a compact subset of the four-dimensional real space $R^{4}$.

The properties of the two-parameter Mittag-Leffler function and the model (7) have been studied by many authors [1,2,3,4,5]. The function $E_{\kappa ,\mu }\left(x\right)$ (6) is completely monotonic on the negative real axis for 0<κ≤1 and μ≥κ, i.e., the function $E_{\kappa ,\mu }\left(-x\right)$ is completely monotonic for x>0, Ref. [4] (Equation (E.32)). Whence, since $t_{0}>0$, by virtue of (6), for any t∈T, and any g∈G, we have

(12) $$E_{\alpha -\beta ,1-\beta }\left(-{\left(\frac{t}{\tau_{r}}\right)}^{\alpha -\beta }\right)<E_{\alpha -\beta ,1-\beta }\left(0\right)=\frac{1}{\Gamma \left(1-\beta \right)}\leq 1.$$

Let us introduce the function [4] (Equation (E.53))

(13) $$e_{\kappa ,\mu }\left(x;\lambda \right)=x^{\mu -1}E_{\kappa ,\mu }\left(-\lambda x^{\kappa }\right),$$

which, comparing (7) and (13), enables describing the relaxation modulus $G_{M}\left(t,g\right)$ (7) in compact form as follows

(14) $$G_{M}\left(t,g\right)=G_{e}{\tau_{r}}^{\beta }e_{\alpha -\beta ,1-\beta }\left(t;{\tau_{r}}^{\beta -\alpha }\right).$$

The function $e_{\kappa ,\mu }\left(x;\lambda \right)$ (13) is known to play a crucial role in many problems of fractional calculus [4] (p. 372) because it has many excellent and useful properties; some of them were used in this paper. The function $e_{\kappa ,\mu }\left(x;\lambda \right)$ is completely monotonic for x>0 when 0<κ≤μ≤1 whenever the parameter λ>0 [4] (p. 373) as the product of two completely monotonic functions, which by (14) implies the complete monotonicity of the relaxation modulus model $G_{M}\left(t,g\right)$ for t>0 whenever 0≤β<α≤1. This means, in particular, that for t>0 and g∈G, such that 0<β<α≤1, the positive definite model $G_{M}\left(t,g\right)$ (7) monotonically decreases with increasing t>0. Therefore, for any t>0 and any g∈G, such that 0<β<α≤1, in view of (12)–(14), we have

(15) $$\left|G_{M}\left(t,g\right)\right|\leq G_{e}{\tau_{r}}^{\beta }t_{0}^{-\beta }E_{\alpha -\beta ,1-\beta }\left(-{\left(\frac{t_{0}}{\tau_{r}}\right)}^{\alpha -\beta }\right)\leq G_{emax}m_{0},$$

where $m_{0}$ is defined below by the sequence of inequalities valid for any t∈T and any g∈G

(16) $${\left(\frac{t}{\tau_{r}}\right)}^{-\beta }\leq {\left(\frac{\tau_{rmax}}{t_{0}}\right)}^{\beta }\leq \frac{\tau_{rmax}}{t_{0}}=m_{0},$$

where $t_{0}>0$.

For the case α=β, the relaxation modulus $G_{M}\left(t,g\right)$ (10) is also a completely monotonic function of the time for t>0, which in view of (16) is uniformly bounded for t∈T and g∈G by $G_{emax}m_{0}/2$.

Therefore, there exists a positive constant $M_{1}=G_{emax}m_{0}$ such that

(17) $$\underset{t\in T,g\in G}{\sup}\left|G_{M}\left(t,g\right)\right|\leq M_{1}<\infty ,$$

i.e., the modulus $G_{M}\left(t,g\right)$ is uniformly bounded on the set T×G.

Inequality (17) combined with Assumption 1 implies the upper bound

(18) $$\underset{t\in T,g\in G}{\sup}\left|G\left(t\right)-G_{M}\left(t,g\right)\right|\leq M+M_{1}<\infty .$$

The Lipschitz continuity of the model $G_{M}\left(t,g\right)$ with respect to parameter g, which is not obvious, in particular, with respect to non-integer orders of fractional derivatives, is fundamental to guarantee the convergence of the optimal models for the applied here experiment randomization. Therefore, before the identification concept and the respective algorithm are presented, the Lipschitz property of the mapping $G_{M}\left(t,g\right)$ (7) will be proved, as summarized in the following theorems. The quite tedious proofs are moved into Appendix A.1.

### 2.3. Lipschitz Continuity of FMM with Respect to Model Parameters

Due to the relation between the parameter α and β, let us consider two cases separately when (a) β<α and (b) β=α. Therefore, the set of admissible model parameters G (11) is decomposed on two disjoint subsets:

(19) $$G_{1}=\left\{g:\beta_{0}\leq \;\beta <\alpha \leq 1;\;G_{emin}\leq E\leq G_{emax};\tau_{rmin}\leq \tau_{r}\leq \tau_{rmax}\right\}$$

and

(20) $$G_{2}=\left\{g:\beta_{0}\leq \;\beta =\alpha \leq 1;\;G_{emin}\leq E\leq G_{emax};\tau_{rmin}\leq \tau_{r}\leq \tau_{rmax}\right\},$$

in which the relaxation modulus $G_{M}\left(t,g\right)$ is described by the formulas (7) and (10), respectively. The bounded set $G_{1}$ is non-closed, i.e., the compactness property of the set G (11) is lost here, while $G_{2}$ is compact.

The following spectral representation derived in [47]

(21) $$G_{M}\left(t,g\right)=G_{e}\frac{\tau_{r}^{\alpha }}{\pi }\int_{0}^{\infty }\frac{{\left(\tau_{r}v\right)}^{\alpha -\beta }\sin\left(\pi \beta \right)+\sin\left(\pi \alpha \right)}{{\left(\tau_{r}v\right)}^{2\left(\alpha -\beta \right)}+2{\left(\tau_{r}v\right)}^{\alpha -\beta }\cos\left[\pi \left(\alpha -\beta \right)\right]+1}v^{\alpha -1}e^{-tv}dv,$$

which results from the known spectral representation of the two-parameter Mittag-Leffler function [1] (Theorem 4.18, Equations (4.7.17) and (4.7.15)) and is valid for 0<β<α≤1, will be used for $g\in G_{1}$. Applying the differential approach in Appendix A.1, the next result is proved.

**Theorem** **1.** *Let* $G_{1}$ *defined by (19) be the set of the fractional Maxwell model admissible parameters. Then, the relaxation modulus* $G_{M}\left(t,g\right)$ *(7) of the FMM (2) is continuous and differentiable with respect to* g *(8) for any time* t∈T *and*

(22) $$\underset{t\in T,g\in G_{1}}{\sup}{\left\|\nabla_{g}G_{M}\left(t,g\right)\right\|}_{2}\leq M_{2}<\infty ,$$

*where* $\nabla_{g}G_{M}\left(t,g\right)$ *denotes the gradient of the function* $G_{M}\left(t,g\right)$ *with respect to the vector* g*; here,* ${\left\|\cdot \right\|}_{2}$ *is the Euclidean norm in the space* $R^{4}$.

The above theorem means, in particular, that for an arbitrary small positive $\beta_{0}$, the mapping $G_{M}:T\times G_{1}\to R$ defined according to Equation (7) is, uniformly with respect to the time t∈T, a Lipschitz continuous function of the vector of model parameters g with Lipschitz constant $M_{2}$.

In the case (b) β=α, for the set of model parameters $G_{2}$ (20), the FMM (2) is described by the power-law relaxation modulus $G_{M}\left(t,g\right)$ (10) and the absolute boundness of the gradient $\nabla_{g}G_{M}\left(t,g\right)$ is resolved by the next result proved in Appendix A.2.

**Theorem** **2.** *Let* $G_{2}$*, defined by (20), be the set of the fractional Maxwell model admissible parameters with equal parameters*α *and* β*. Then, the relaxation modulus* $G_{M}\left(t,g\right)$ *(10) of the model (9) is continuous and differentiable with respect to* g *(8) for any time* t∈T *and*

(23) $$\underset{t\in T,g\in G_{2}}{\sup}{\left\|\nabla_{g}G_{M}\left(t,g\right)\right\|}_{2}\leq M_{3}<\infty .$$

From the proofs of the above theorems, especially from the nonnegative definiteness of the derivatives $\frac{\partial G_{M}\left(t,g\right)}{\partial E}$ (A5), $\frac{\partial G_{M}\left(t,g\right)}{\partial \tau_{r}}$ (A6) and the two last elements of the gradient $\nabla_{g}G_{M}\left(t,g\right)$ (A53), the following property is derived.

**Property** **1.** *Let* G *defined by (11) be the set of the FMM (2) admissible parameters. Then, for any fixed time* t∈T*, the relaxation modulus* $G_{M}\left(t,g\right)$ *described by (7) or (10) monotonically increases with increasing parameters* $G_{e}$ *and* $\tau_{r}$ *and other parameters being fixed, i.e., the greater parameters* $G_{e}$ *and* $\tau_{r}$ *are, the greater the relaxation modulus* $G_{M}\left(t,g\right)$ *is for the given* t∈T.

### 2.4. Relaxation Modulus Measurements

Following [33,35], let $T_{1},\ldots ,T_{N}$ be independent random variables with a common probability density function $\rho \left(t\right)$; T is the support of $\rho \left(t\right)$. Then, let $G_{i}=G\left(T_{i}\right)$ be the related relaxation modulus of the material for i=1,…,N. Let ${\bar{G}}_{i}$ denote their measurements corrupted by additive noise $Z_{i}$, i.e., ${\bar{G}}_{i}=G_{i}+Z_{i}$, recorded in the stress relaxation experiment [44,46,48].

The two assumptions concerning the measurement noises are taken (compare Assumptions 5 and 6 in [33]) as follows:

**Assumption** **2.** *The measurement noise* $\left\{Z_{i}\right\}$ *is a time-independent, i.e., independent of the variables* $\left\{T_{i}\right\}$*, sequence of independent identically distributed (i.i.d.) random variables with zero mean* $E\left[Z_{i}\right]=0$ *and a common finite variance* $E\left[Z_{i}^{2}\right]=\sigma^{2}<\infty$.

**Assumption** **3.** *The measurement noises* $Z_{i}$ *are bounded by* δ*, i.e.,* $\left|Z_{i}\right|\leq \delta <\infty$ *for* i=1,…,N.

Both the above assumptions and Assumption 1, concerning the real relaxation modulus, are natural in the context of the relaxation modulus identification [33].

### 2.5. Identification Problem

FMM identification involves selecting from a given class of models defined by (7) and (10) the model that best fits the measurement data. Suppose an identification experiment resulted in a set of measurements $\left\{\bar{G}\left(T_{i}\right)=G\left(T_{i}\right)+Z_{i}\right\}$ at the sampling times $T_{i}\geq t_{0}>0$, i=1,…,N. The mean-squares index

(24) $$Q_{N}\left(g\right)=\frac{1}{N}\sum_{i=1}^{N}{\left[\bar{G}\left(T_{i}\right)-G_{M}\left(T_{i},g\right)\right]}^{2},$$

is taken as a measure of the FMM model accuracy. Here, the lower index denotes the number of measurements. Then, the problem of the optimal model identification consists of the solution of the minimization task

(25) $$\underset{g\in G}{min}\;\;Q_{N}\left(g\right)=Q_{N}\left(g_{N}^{*}\right),$$

where $g_{N}^{*}$ is the optimal model parameter. Since, due to the continuity of the model $G_{M}\left(t,g\right)$ with respect to the parameter g, the index $Q_{N}\left(g\right)$ is a continuous function of g and the set of admissible parameters G (11) is compact, the existence of the solution to the optimization problem (25) immediately results from the Weierstrass theorem about the extreme of continuous function on the compact set [49]. Since the minimum $g_{N}^{*}$ can be not unique, let $G_{N}^{*}$ denote the set of vectors $g_{N}^{*}$ that solve the optimization task (25).

The parameters $g_{N}^{*}$ of the identified relaxation modulus model $G_{M}\left(t,g_{N}^{*}\right)$ are dependent on the measurement data, in particular, on the sampling instants $T_{i}$. To make the model independent of specific sampling instants $T_{i}$, the optimal sampling points-independent approximation problem is stated in the following subsection.

### 2.6. The Optimal FMM

Let us consider the following problem of determining such an FMM that minimizes the global approximation error:

(26) $$Q\left(g\right)=\int_{T}{\left[G\left(t\right)-G_{M}\left(t,g\right)\right]}^{2}\rho \left(t\right)dt,$$

where the selected weight function, such that $0\leq \rho \left(t\right)\leq M_{0}<\infty$, is a density on the set T, i.e., $\int_{T}\rho \left(t\right)dt=1$.

The integral (26) is absolutely integrable, uniformly on G, both for the bounded or unbounded domain T as the product of a function ${\left[G\left(t\right)-G_{M}\left(t,g\right)\right]}^{2}$, in view of (18) bounded uniformly for $\left(t,g\right)\in T\times G$, and absolutely integrable function $\rho \left(t\right)$. Therefore, the integral (26) is well defined for any g∈G**.**

The problem of the optimal approximation of the real modulus $G\left(t\right)$ within the class of the fractional Maxwell models relies on determining the parameter $g^{*}$ that minimizes $Q\left(g\right)$ over the set G, i.e., in solving optimization task

(27) $$\underset{g\in G}{min}\;Q\left(g\right)=Q\left(g^{*}\right).$$

Due to continuity of $G_{M}\left(t,g\right)$ with respect to the vector g, the index $Q\left(g\right)$ (5) is a continuous function of g, and thus, the existence of the solution $g^{*}$ follows from the previously mentioned Weierstrass theorem concerning the extreme of continuous function on the compact set. Let the set of model parameters $g^{*}$ solving (27) be denoted by $G^{*}$. Any parameter $g^{*}{\in G}^{*}$ does not depend on the specific time instants applied in the experiment.

## 3. Results and Discussion

In this section, the analysis of the asymptotic properties of the identified fractional Maxwell model, when the number of measurements tend to infinity, is conducted. The rate of the convergence of this model to the optimal FMM, which does not depend on the experiment data, is studied. The resulting identification algorithm is outlined. Next, the analytically proven properties of the identification method are verified by numerical simulations and studies. Two example materials are simulated. In the first, the “real” material is described by a unimodal Gauss-like relaxation spectrum [37,38,39] with short relaxation times and the Baumgaertel, Schausberger and Winter (BSW) spectrum [40,41] with long relaxation times. Such models are used to describe the rheological properties of various materials, especially polymers and biopolymers. Based on the noise-corrupted data from the simulated randomized stress relaxation experiment, the optimal FMM models are determined. The asymptotic properties and noise robustness have been studied.

### 3.1. Convergence

The empirical index $Q_{N}\left(g\right)$ (24) can be obtained by the replacement of the integral in $Q\left(g\right)$ (26) with the finite mean sum of squares, which is clear from a practical point of view. For i=1,…,N, by Assumption 2, the expected value is

$$E{\left[G\left(T_{i}\right)+Z_{i}-G_{M}\left(T_{i},g\right)\right]}^{2}=Q\left(g\right)+\sigma^{2},$$

whence, by (24), the expected value is

(28) $$EQ_{N}\left(g\right)=Q\left(g\right)+\sigma^{2}.$$

To investigate the stochastic-type asymptotic properties of the empirical identification task given by (25), some properties derived in [35] will be used. Note, that Assumptions A1–A3 from [35], concerning the compactness of the set of model admissible parameters, continuity, differentiability and Lipshitzness of the model are satisfied here. Taken above, Assumption 2 is identical with Assumption A5 in [35], while property (18) also means that Assumption A4 from [35] is satisfied, i.e., all the assumptions from [35] hold here.

By (28), Property 2 from [35] implies the next result.

**Property** **2.** *When Assumptions 1 and 2 are satisfied, then*

(29) $$\underset{g\in G}{sup}\;\;\left|\left[Q\left(g\right)+\sigma^{2}\right]-Q_{N}\left(g\right)\right|\to 0\;\;w.p.1\;\;as\;\;N\to \infty ,$$

*where* w.p.1 *means “with probability one”.*

By (28) and (29), the empirical identification index $Q_{N}\left(g\right)$ (24) is arbitrarily close to its expected value, uniformly in g over the set G. In consequence, the model parameter $g_{N}^{*}$ solving empirical identification task (25) can be related to the parameter $g^{*}$ that solves the sampling points-independent minimization task (27). From the uniform in g∈G convergence of the index $Q_{N}\left(g\right)$ in (29), we conclude immediately the main result of this subsection, c.f., Assertion in [35] or Equation (3.5) in [34].

**Property** **3.** *Assume that Assumptions 1 and 2 hold,* $T_{1},\ldots ,T_{N}$ *are independently and randomly selected from* T*, each according to the distribution with probability density function* $\rho \left(t\right)$ *. If the solutions to the minimization problems (25) and (27) are unique, then*

(30) $$g_{N}^{*}\to g^{*}\;w.p.1\;\;as\;\;N\to \infty$$

*and*

(31) $$G_{M}\left(t,g_{N}^{*}\right)\to G_{M}\left(t,g^{*}\right)\;w.p.1\;\;as\;\;N\to \infty .$$

*for all* t∈T *. If the minimization problems (25) and (27) do not have unique solutions, then for any convergent subsequence of the sequence* $\left\{g_{N}^{*}\right\}$ *, where* $g_{N}^{*}\in G_{N}^{*}$ *,*

(32) $$g_{N}^{*}\to G^{*}\;w.p.1\;\;as\;\;N\to \infty$$

*and for any* t∈T *and some* $g^{*}\in G^{*}$ ***,*** *the convergence in (31) holds.*

The existence of a convergent subsequence of $\left\{g_{N}^{*}\right\}$ so that the asymptotic property (32) holds results directly from the compactness of G (11). Therefore, under Assumptions 1 and 2, the optimal parameter $g_{N}^{*}$ of the FMM is a strongly consistent estimate of some parameter $g^{*}\in G^{*}$.

Since, by Theorems 1 and 2, the model $G_{M}\left(t,g\right)$ is Lipschitz on G uniformly in t∈T, then the almost-sure convergence of $g_{N}^{*}$ to $g^{*}$ in (30) implies that, c.f., (Ref. [35]: Remark 2):

(33) $$\underset{t\in T}{sup}\;\;\left|G_{M}\left(t,g_{N}^{*}\right)-G_{M}\left(t,g^{*}\right)\right|\to 0\;\;w.p.1\;\;as\;\;N\to \infty .$$

i.e., that $G_{M}\left(t,g_{N}^{*}\right)$ is a strongly consistent estimate of the optimal FMM $G_{M}\left(t,g^{*}\right)$, uniformly on T.

Concluding, when Assumptions 1 and 2 are satisfied, the arbitrarily fine approximation of the FMM with the optimal parameter $g^{*}$ can be determined (almost everywhere) as the number of measurements N grow enough, even if the real description of the material modulus is fully unknown.

### 3.2. Exponential Rate of Convergence

Analyzing the convergence in (30) and (32), the question immediately arises of how fast $g_{N}^{*}$ tends to some $g^{*}\in G^{*}$ as N grows large. As in [35], the distance between the model parameters $g_{N}^{*}$ and $g^{*}$ will be evaluated by means of the integral identification index $Q\left(g\right)$ (26), i.e., in the sense of the difference $\left|Q\left(g^{*}\right)-Q\left(g_{N}^{*}\right)\right|$. For this purpose, it will be checked how fast, for a given small ε>0, the probability $P\left\{\left|Q\left(g^{*}\right)-Q\left(g_{N}^{*}\right)\right|\geq \varepsilon \right\}$ tends to zero, as N increases. From the well-known Hoeffding’s inequality [50], the upper bound of this probability can be derived, analogous to inequality (15) in [35] or inequality (22) in [33] (for details, see Appendix A.1 in [33]):

(34) $$P\left\{\left|Q\left(g^{*}\right)-Q\left(g_{N}^{*}\right)\right|\geq \varepsilon \right\}\leq 2exp\left(\frac{-N\varepsilon^{2}}{8{\hat{M}}^{2}}\right),$$

for any ε>0, where

(35) $$\hat{M}={2\left(M+M_{1}\right)}^{2}+\sigma^{2}+\delta^{2}+2\left(M+M_{1}\right)\delta ,$$

with the constants M and $M_{1}$ defined in Assumption 1 and Equation (17), respectively, the noises’ variance $\sigma^{2}$ and upper bound δ are introduced by Assumptions 2 and 3.

The inequality (34) describes the influence of the number of measurements N and the noises’ ”strength” on the rate of convergence. For ε being fixed, the bounds for $P\left\{\left|Q\left(g^{*}\right)-Q\left(g_{N}^{*}\right)\right|\geq \varepsilon \right\}$ decrease exponentially to zero as N increases. The convergence rate is the higher, the lower is $\hat{M}$ (35). In particular, a quick inspection of (35) shows that for stronger measurement noises, the rate of convergence is reduced. Larger δ and σ yield a greater decrease in the rate. This is as expected, since with large disturbances, the measurements are not very adequate. Simultaneously, the larger $M+M_{1}$, i.e., in view of the estimation (18), the greater the discrepancy between the real modulus and the FMM, the worse the convergence.

### 3.3. Identification Algorithm

In view of the convergence properties (30), (31) the computation of the parameter $g_{N}^{*}$ approximation the parameter $g^{*}$ of the optimal FMM requires the next steps:

1. Select randomly from the set T the sampling times $t_{1},\ldots ,t_{N}$, choosing each $t_{i}$ independently, according to the probability distribution of the density $\rho \left(t\right)$ defined given by the weight function in the integral $Q\left(g\right)$ (26).
2. Conduct the stress relaxation experiment [44,46,48], measure and store the measurements $\left\{{\bar{G}}_{i}\right\}$ of the relaxation modulus for the selected time instants $t_{i}$, i=1,…,N.
3. Solve the identification optimization task (25) and compute the identified model parameter $g_{N}^{*}$.
4. Put $\bar{N}=N$ and $g_{\bar{N}}^{*}=g_{N}^{*}$. To extend the set of experiment data, select new $N\gg \bar{N}$.
5. Repeat Steps 1–3 for a new N, that is, randomly choose new sampling times, conduct the rheological experiment once more for a new sample of the material and determine the next $g_{N}^{*}$.
6. Examine if ${\left\|g_{\bar{N}}^{*}-g_{N}^{*}\right\|}_{2}<\varepsilon$, where ε is a small positive number, to check if $g_{\bar{N}}^{*}$ is an adequate approximation of $g^{*}$. If yes, stop the scheme and take $g_{\bar{N}}^{*}$ as the approximate value of $g^{*}$. Otherwise, go again to Step 4.

**Remark** **1.** *A less restrictive testing regarding whether* $\left|Q_{\bar{N}}\left(g_{\bar{N}}^{*}\right)-Q_{N}\left(g_{N}^{*}\right)\right|<\varepsilon$ *holds can be used as an alternative for the stopping rule from Step 6. Both types of stopping rules are commonly used in numerical optimization techniques.*

### 3.4. Numerical Studies

The results of the numerical studies are concerned with the asymptotic properties of the determined optimal FMM and the influence of the measurement noises on this model. Apart from the theoretical analysis above, these simulation studies make it possible to show the respectability and effectiveness of the method developed for FMM identification.

Firstly, it is assumed that the rheological properties of the material are described by the Gaussian-like distribution of the relaxation spectrum, which were used to represent the viscoelastic properties of numerous materials, e.g., polyacrylamide gels [48], native starch gels [38], glass [39], poly(methyl methacrylate) [37], polyethylene [51] and carboxymethylcellulose (CMC) [52]. The spectra of various biopolymers determined by many researchers are Gaussian in nature, for example, cold gel-like emulsions stabilized with bovine gelatin [53], fresh egg white-hydrocolloids [52], some (wheat, potato, corn and banana) native starch gels [38], xanthan gum water solution [52] and wood [54,55].

Next, it is assumed that the material is modeled by the Baumgaertel, Schausberger and Winter (BSW) spectrum [40,41], which was used to describe the viscoelasticity of various polymers; for example, polydisperse polymer melts [42,43], polymethylmethacrylate (PMMA) [56], polybutadiene (PBD) [56] and polymer pelts [57].

The “real” material and the FMM model were simulated in Matlab R2023b, The Mathworks, Inc., Natick, MA, USA. Functions MLFFIT2 [58] and MLF [59], provided by Podlubny, were used for the FMM simulation and numerical solution of the optimal identification tasks.

### 3.5. Material I

Consider the material whose relaxation spectrum is described by the unimodal Gauss-like distribution:

$$H\left(\tau \right)=\vartheta e^{-{\left(\frac{1}{\tau }-m\right)}^{2}/q}/\tau ,$$

where the parameters are as follows [60]: ϑ=31520 Pa·s, $m=0.0912s^{-1}$ and $q=3.25\times 10^{-3}s^{-2}$. The related relaxation modulus is [60]

(36) $$G\left(t\right)=\frac{\sqrt{\pi q}}{2}\vartheta \;e^{\frac{1}{4}t^{2}q-mt}erfc\left(\frac{\frac{1}{2}tq-m}{\sqrt{q}}\right),$$

where the complementary error function $erfc\left(x\right)$ is given by [4] (Equation (C.2))

$$erfc\left(x\right)=\frac{2}{\sqrt{\pi \;}\;}\int_{x}^{\infty }e^{{-z}^{2}}dz.$$

Following [47], for numerical simulations, the time interval $T=\left[0,200\right]$ seconds is chosen. Hence, the weighting function in $Q\left(g\right)$ (26) is $\rho \left(t\right)=\frac{1}{200}s^{-1}$. The elements of the optimal parameter vector $g^{*}$ solving the measurement-independent optimization task (27) are given in Table 1.

The N sampling instants $t_{i}$ for the simulated stress relaxation test were selected randomly according to the uniform distribution on T. A normal distribution with zero mean value and variance $\sigma^{2}$ was applied to the random independent generation of the additive measurement noises $\left\{z_{i}\right\}$. In the noise robustness analysis, the standard deviations $\sigma =2,5,8\;\left[Pa\right]$ were used. In the analysis of the model asymptotic properties, for any σ numbers of measurements, N∈N have been applied, where $N=\left\{50;100;200;500;1000;2000;5000;7000;10,000;12,000;15,000\right\}$.

#### 3.5.1. Asymptotic Properties

Then, for every pair $\left(N,\sigma \right)$, the optimal parameter $g_{N}^{*}$ was determined through solving the minimization task (25). The elements of the vectors $g_{N}^{*}$, the mean square indices $Q_{N}\left(g_{N}^{*}\right)$ and integral $Q\left(g_{N}^{*}\right)$ indices, and the relative percentage errors of the approximation of the measurement-independent parameter $g^{*}$, defined as

(37) $$ERR={\left\|g_{N}^{*}-g^{*}\right\|}_{2}^{2}/{\left\|g^{*}\right\|}_{2}^{2}\cdot 100\%,$$

are given in Table A1, Table A2 and Table A3 for the three standard deviations of the noises. The model approximation error was also estimated via the relative mean error defined as (compare (24))

(38) $$Q_{Nrel}\left(g\right)=\frac{1}{N}\sum_{i=1}^{N}\frac{{\left[\bar{G}\left(T_{i}\right)-G_{M}\left(T_{i},g\right)\right]}^{2}}{{\left[\bar{G}\left(T_{i}\right)\right]}^{2}}.$$

**Figure 2:**
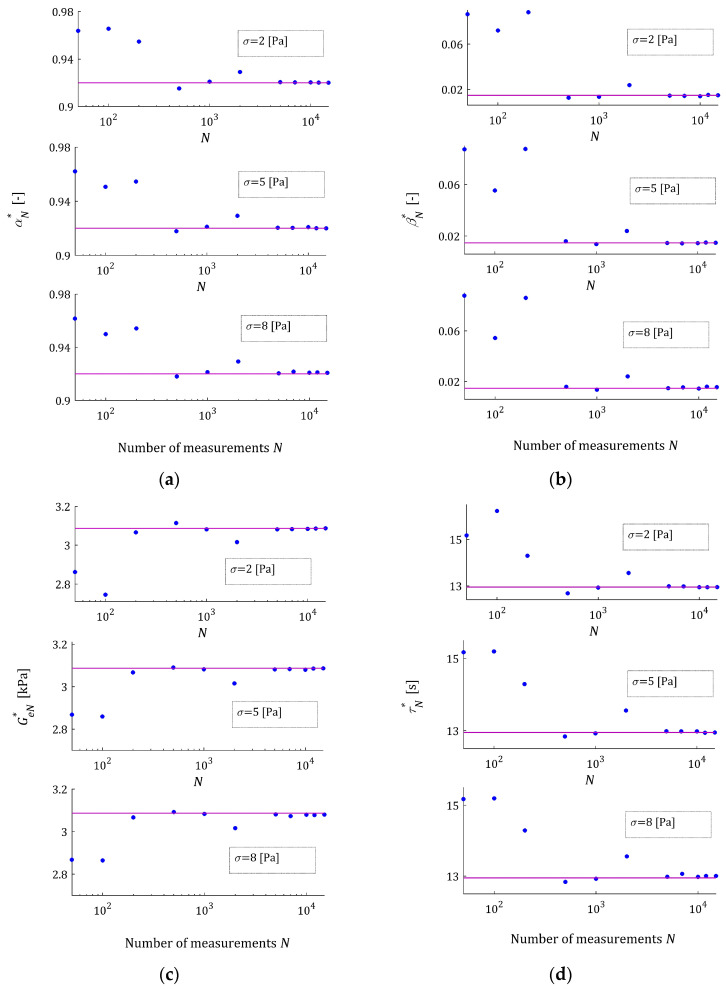
Dependence of the parameters of the FMM approximating the “real” relaxation modulus (36): (**a**) $\alpha_{N}^{*}$; (**b**) $\beta_{N}^{*}$; (**c**) $G_{eN}^{*}$; and (**d**) $\tau_{rN}^{*}$ on the number of measurements N for disturbances $\sigma =2,5,8\;\left[Pa\right]$; the horizontal purple lines are related to the optimal parameters $\alpha^{*}$, $\beta^{*}$, $G_{e}^{*}$ and $\tau_{r}^{*}$ independent on the sampling instants used in the rheological experiment.

**Figure 3:**
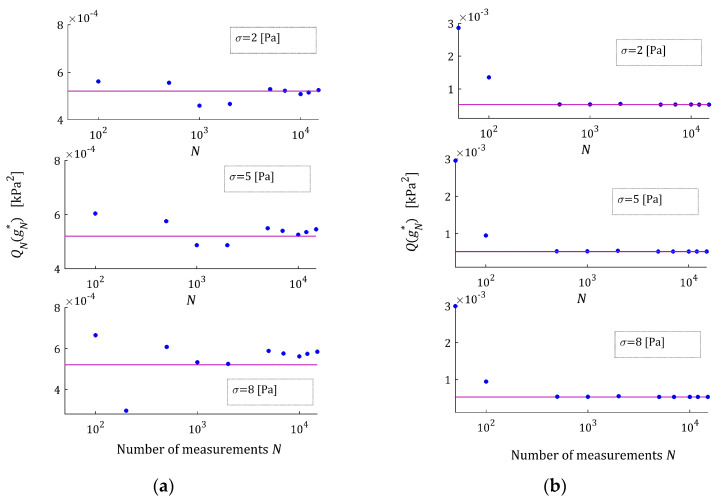
The indices of the “real” relaxation modulus (36) approximation by the FMM: (**a**) the mean-square empirical index $Q_{N}\left(g_{N}^{*}\right)$, Equation (24), (**b**) the integral quadratic sampling instants-independent index $Q\left(g_{N}^{*}\right)$, Equation (26), as the functions of the number of measurements N and noises $\sigma =2,5,8\;\left[Pa\right]$; the horizontal purple lines correspond to the optimal integral index $Q\left(g^{*}\right)$ defined in Equation (27).

The optimal model parameters $g_{N}^{*}$ as the functions of the number of measurements N are illustrated by Figure 2 for the noises of $\sigma =2,5,8\;\left[Pa\right]$. In any subplot, the values of the related parameters of the sampling points-independent model $g^{*}$ are depicted by horizontal purple lines. The asymptotic properties are also illustrated by Figure 3 juxtaposing the empirical index mean-square index $Q_{N}\left(g_{N}^{*}\right)$, Equation (24), and the integral quadratic sampling instants-independent index $Q\left(g_{N}^{*}\right)$, Equation (26), as the functions of N with the index $Q\left(g^{*}\right)$, marked with horizontal lines. In Figure 2 and Figure 3, a logarithmic scale is applied for the horizontal axes. These plots confirm the asymptotic properties of the proposed identification algorithm. The convergence of $g_{N}^{*}$ to the parameter $g^{*}$ is directly translated into the convergence of $Q\left(g_{N}^{*}\right)$ into $Q\left(g^{*}\right)$, especially for N≥5000. The values of the index $Q_{N}\left(g_{N}^{*}\right)$ for N=50, small compared to those for N≥100 (see Table A1, Table A2 and Table A3), result from the good fit of the FMM, whose four parameters are optimally selected in problem (25), to only 50 measurement points. For more measurement points, such a good fit is, generally, impossible whenever the real characteristic does not depend on the pre-assumed class of models. A comparison of Figure 2 and Figure 3b with Figure 3a shows that the impact of stronger noises on the values of the empirical index $Q_{N}\left(g_{N}^{*}\right)$ is much stronger than the impact of the noises on the values of the FMM parameter $g_{N}^{*}$ and, consequently, also on the integral index $Q\left(g_{N}^{*}\right)$, which does not directly depend on the measurement noises. Given Equation (28), this property is natural and fully justified.

**Figure 4:**
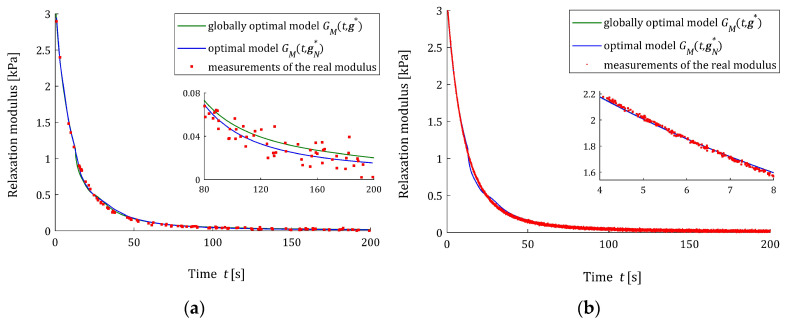
The measurements ${\bar{G}}_{i}$ (red points) of the “real” relaxation modulus (36) and optimal FMM models: sampling points-independent $G_{M}\left(t,g^{*}\right)$ and empirical $G_{M}\left(t,g_{N}^{*}\right)$ for N measurements and normal distribution noises with the standard deviation $\sigma =8\;\left[Pa\right]$: (**a**) N=100; (**b**) N=10,000.

The quality of the real modulus $G\left(t\right)$ approximation by the FMM is illustrated in Figure 4, where the measurements ${\bar{G}}_{i}$ of the real modulus $G\left(t\right)$ fitted by the optimal model $G_{M}\left(t,g_{N}^{*}\right)$ are plotted for the N=100 and N=10,000 measurements and the strongest disturbances; $\sigma =8\;\left[Pa\right]$. Although, for the N=100 measurements, the models $G_{M}\left(t,g_{N}^{*}\right)$ and $G_{M}\left(t,g^{*}\right)$ differ slightly (see small subplot), for the N=10,000 measurements, they are practically identical, which is confirmed by the values of ERR (37) equaling 0.52% for N=100 and equaling only 5.58 × 10^−4^ % for N=10,000 (see Table A3). Even for the strongest noises, the relative errors ERR (37) of the parameters $g^{*}$ and $g_{N}^{*}$ discrepancy is smaller than 0.002% for N≥200. This almost excellent fitting of the experiment data by the model $G_{M}\left(t,g_{N}^{*}\right)$ is confirmed by the values of the relative square model approximation index $Q_{Nrel}\left(g_{N}^{*}\right)$ (38), which for N≥200 and the weakest noises does not exceed 0.015%, while for the strongest noises, it does not exceed 0.28%. For the noises considered, the values of the model fit indices $Q_{N}\left(g_{N}^{*}\right)$ (24) and $Q_{Nrel}\left(g_{N}^{*}\right)$ (38) and the integral quadratic index $Q\left(g_{N}^{*}\right)$ (26) indicate an excellent fit of the model to the experiment data and the fast convergence of $g_{N}^{*}$ to $g^{*}$ as N tends to infinity; compare Table A1, Table A2 and Table A3.

#### 3.5.2. Noise Robustness

To examine the effect of the measurement noises, for every pair $\left(N,\sigma \right)$, the simulated experiment was repeated n=50 times. In each experiment repetition, the measurement noises $\left\{z_{i}\right\}$ were generated independently and randomly with a normal distribution, with a zero mean value and variance $\sigma^{2}$.

Having in mind the definition of the index $Q_{Nrel}\left(g\right)$ (38), for the n-element sample, the mean relative relaxation modulus approximation error was determined as follows:

(39) $$ERRQ_{Nrel}=\frac{1}{n}\sum_{j=1}^{n}Q_{Nrel}\left(g_{N,j}^{*}\right),$$

for any pair $\left(N,\sigma \right)$, where the vector of the optimal FMM parameters $g_{N,j}^{*}$ was computed for *j*-th experiment repetition, j=1,…,n.

For the true relaxation modulus approximation, the mean optimal integral error

(40) $$ERRQ=\frac{1}{n}\sum_{j=1}^{n}Q\left(g_{N,j}^{*}\right)$$

was determined for every pair $\left(N,\sigma \right)$.

The distance between the vector $g_{N,j}^{*}$ and the measurement-independent vector $g^{*}$ for the n element sample was estimated by the mean relative error defined as follows (compare ERR (37)):

(41) $$MERR=\frac{1}{n}\sum_{j=1}^{n}{\left\|g_{N,j}^{*}-g^{*}\right\|}_{2}^{2}/{\left\|g^{*}\right\|}_{2}^{2}\cdot 100\%.$$

**Figure 5:**
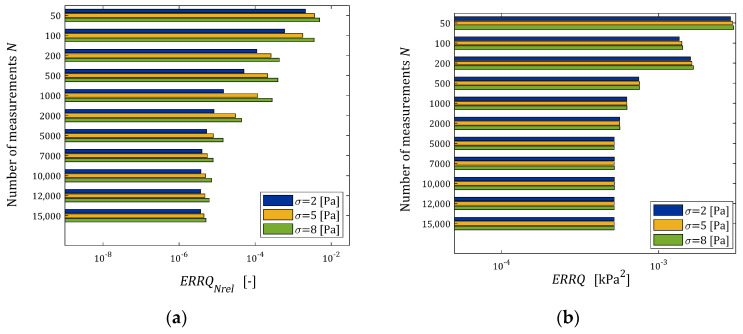
Dependence of the mean indices of the “real” relaxation modulus $G\left(t\right)$ (36) optimal approximation by the FMM: (**a**) relative empirical error $ERRQ_{Nrel}$ (39), (**b**) integral error ERRQ (40) on the number of measurements N and the noises’ standard deviations σ.

**Figure 6:**
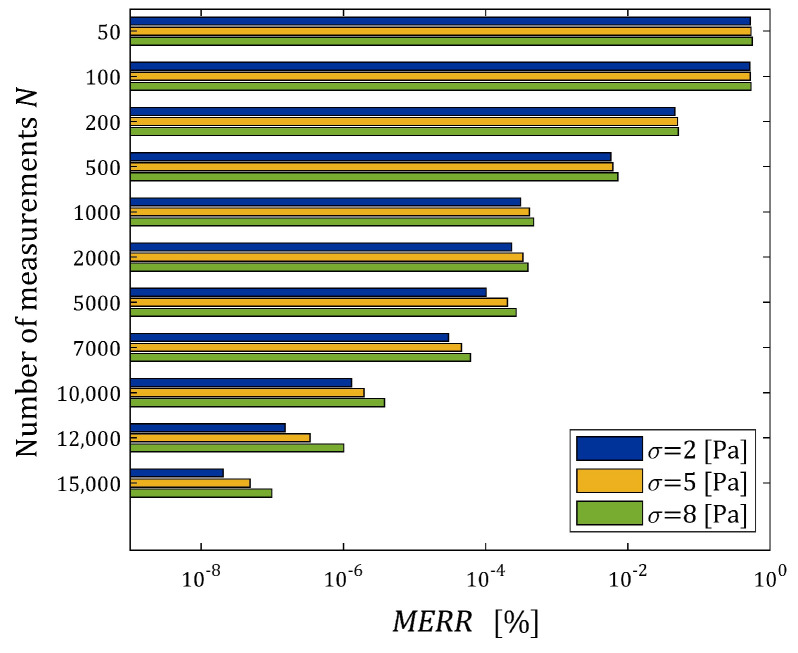
Dependence of the mean relative error MERR (41) between the optimal parameters $g_{N}^{*}$ and $g^{*}$ of the FMM approximating the “real” relaxation modulus $G\left(t\right)$ (36) on the number of measurements N and the noises’ standard deviation σ.

The indices $ERRQ_{Nrela}$ (39) and ERRQ (40), as the functions of N and σ, are depicted in the bar in Figure 5, while the index MERR (41) is shown in Figure 6.

From Figure 5b, it is seen that for N>2000, the number of measurements do not essentially affect the integral index ERRQ, either for weak or strong noises, while both the empirical index $ERRQ_{Nrel}$ and mean relative error MERR decrease exponentially with the increasing number of measurements, which confirms the analytical analysis performed above. The MERR index is of order 0.55% for N=100, it does not exceed $10^{-3}\;\%$ for N≥1000 and is smaller than ${5\times 10}^{-5}\;\%$ even for the strongest disturbances. This, practically, means determining the sampling points-independent parameter $g^{*}$. The algorithm ensures the very good quality of the measurement approximation even for large noises. The values of the relative relaxation modulus approximation error $ERRQ_{Nrel}$, which due to the “real” modulus model difference is lower bounded by ${3.191\times 10}^{-4}$%, already for N≥100 measurements do not exceed 0.35%, and for N≥1000, fall below 0.028%. The course of the mean integral sampling points-independent index ERRQ (40) as the function of N indicates the asymptotic independence of the model from the sampling points, especially for N≥5000.

### 3.6. Material II

Consider the material described by the empirical spectrum of relaxation times τ introduced by Baumgaertel, Schausberger and Winter [40,41],

(42) $$H\left(\tau \right)=\left\{\beta_{1}{\left(\frac{\tau }{\tau_{c}}\right)}^{\rho_{1}}+\beta_{2}{\left(\frac{\tau }{\tau_{c}}\right)}^{\rho_{2}}\right\}e^{-\frac{\tau }{\tau_{max}}},$$

which is known to effectively describe polydisperse polymer melts [42,43], with the coefficients [43,47,61] as follows: $\beta_{1}=6.276\times 10^{-2}\;MPa$, $\beta_{2}=0.127\;MPa$, $\tau_{c}=2.481\;s$, $\tau_{max}=2.564\times 10^{4}\;s$, $\rho_{1}=0.25$ and $\rho_{2}=-0.5$. The spectrum $H\left(\tau \right)$ uniquely defines the relaxation modulus $G\left(t\right)$ by the following integral [44]:

(43) $$G\left(t\right)=\int_{0}^{\infty }\frac{H\left(\tau \right)}{\tau }e^{-t/\tau }d\tau .$$

Following [47], the time interval $T=\left[0,2000\right]$ seconds is taken for the experiment simulations; the weighting function is $\rho \left(t\right)=\frac{1}{2000}s^{-1}$. The elements of the optimal parameter vector $g^{*}$, which solve the measurement-independent optimization task (27), are given in Table 2.

As previously described, in the simulations, the sampling points $t_{i}$ were randomly selected according to the uniform distribution on T. The standard deviations $\sigma =3,6,8\;\left[kPa\right]$ of the random normally distributed noises $\left\{z_{i}\right\}$ combined with the number of measurements N∈N were used for the analysis of the model asymptotic properties.

#### 3.6.1. Asymptotic Properties

**Figure 7:**
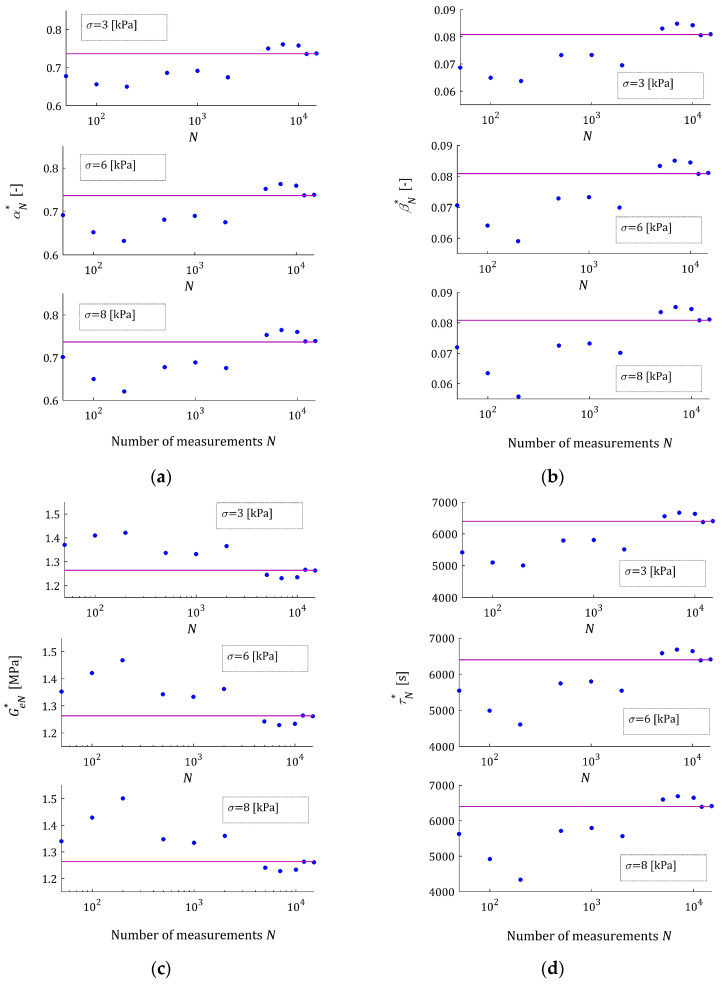
The parameters of the FMM approximating the relaxation modulus (43) of material described by the BSW relaxation spectrum (42): (**a**) $\alpha_{N}^{*}$; (**b**) $\beta_{N}^{*}$; (**c**) $G_{eN}^{*}$; and (**d**) $\tau_{rN}^{*}$ as the functions of the number of measurements N for noises $\sigma =3,6,8\;\left[kPa\right]$; the horizontal purple lines correspond to optimal model parameters $\alpha^{*}$, $\beta^{*}$, $G_{e}^{*}$ and $\tau_{r}^{*}$ being independent on the sampling instants used in the experiment.

**Figure 8:**
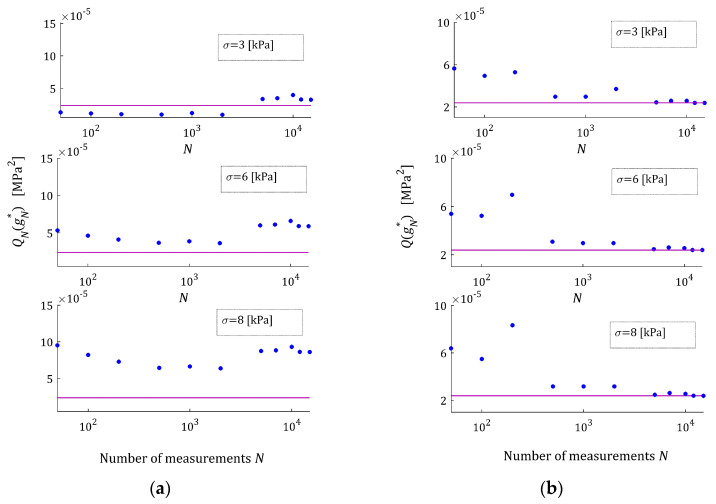
The indices of the BSW relaxation modulus (42), (43) approximation by the FMM: (**a**) the mean-square empirical index $Q_{N}\left(g_{N}^{*}\right)$, Equation (24), (**b**) the integral quadratic sampling instants-independent index $Q\left(g_{N}^{*}\right)$, Equation (26), as the functions of the number of measurements N for the noises $\sigma =2,5,8\;\left[Pa\right]$; the horizontal purple lines correspond to the optimal integral index $Q\left(g^{*}\right)$ defined in Equation (27).

For every pair $\left(N,\sigma \right)$, the elements of the optimal model parameter $g_{N}^{*}$, the empirical $Q_{N}\left(g_{N}^{*}\right)$, $Q_{Nrel}\left(g_{N}^{*}\right)$ and integral $Q\left(g_{N}^{*}\right)$ indices and the relative percentage errors ERR (37) are given in Table A4, Table A5 and Table A6 in Appendix B. The dependence of the optimal model parameters $g_{N}^{*}$ on the number of measurements N for the noises of $\sigma =3,6,8\;\left[kPa\right]$ are illustrated by Figure 7. Figure 8 illustrates the empirical $Q_{N}\left(g_{N}^{*}\right)$ and integral $Q\left(g_{N}^{*}\right)$ indices as the functions of N; the value of $Q\left(g^{*}\right)$ is marked with purple horizontal lines. These plots confirm the asymptotic properties of the proposed identification algorithm. Figure 8a shows the impact of noises on the values of the empirical index $Q_{N}\left(g_{N}^{*}\right)$.

**Figure 9:**
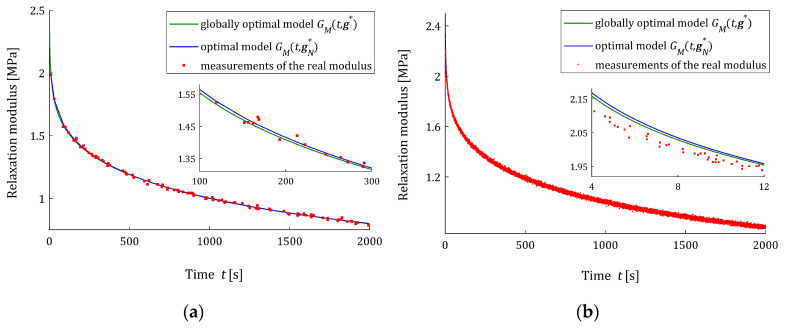
The measurements ${\bar{G}}_{i}$ (red points) of the real relaxation modulus (43) of the material described by the BSW spectrum (42) and the fractional Maxwell optimal models: sampling points-independent $G_{M}\left(t,g^{*}\right)$ and empirical $G_{M}\left(t,g_{N}^{*}\right)$ for N measurements and additive random normally distributed noises with standard deviation $=8\;\left[Pa\right]$ and zero mean value: (**a**) N=100; (**b**) N=10,000.

The approximation of the real modulus $G\left(t\right)$ by the FMM is illustrated in Figure 9, where the measurements ${\bar{G}}_{i}$ of the real modulus $G\left(t\right)$ along with optimal models $G_{M}\left(t,g_{N}^{*}\right)$ and $G_{M}\left(t,g^{*}\right)$ are plotted for the N=100 and N=10,000 measurements and the strongest noises $\sigma =8\;\left[Pa\right]$. However, for N=100, the model parameter error ERR=5.35%, while for N=10,000, we have ERR=0.15%; both for N=100 and N=10,000, the models $G_{M}\left(t,g_{N}^{*}\right)$ and $G_{M}\left(t,g^{*}\right)$ differ slightly and the respective empirical indices are $Q_{Nrel}\left(g_{N}^{*}\right)=$3.33 × 10^−4^ % and $Q_{Nrel}\left(g_{N}^{*}\right)=$2.0 × 10^−7^%, respectively.

#### 3.6.2. Noise Robustness

**Figure 10:**
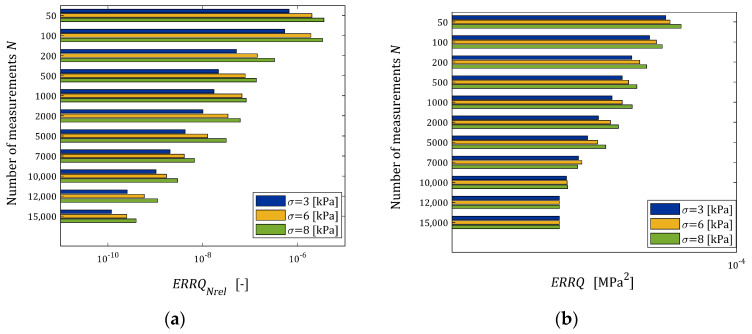
Dependence of the mean indices of the “real” BSW relaxation modulus (42), (43) approximation by the FMM: (**a**) the mean relative empirical error $ERRQ_{Nrel}$ (39), (**b**) the mean optimal sampling points-independent integral error ERRQ (40) on the number of measurements N and the noises’ standard deviation σ.

**Figure 11:**
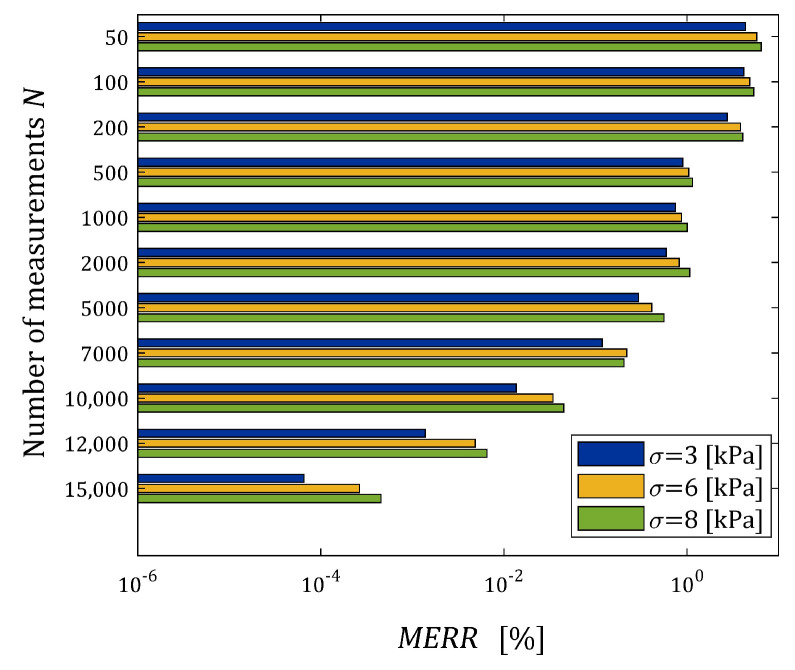
Dependence of the mean relative error MERR (41) between the parameters $g^{*}$ and $g_{N}^{*}$ of the FMM approximating the “real” BSW relaxation modulus (42), (43) on the number of measurements N and the noises’ standard deviation σ.

For every pair $\left(N,\sigma \right)$, the simulated experiment was repeated n=50 times. The mean relative relaxation modulus approximation error $ERRQ_{Nrel}$ (39), the mean optimal integral error ERRQ (40) and the mean relative error of the parameter $g^{*}$ approximation MERR (41) were determined. The indices $ERRQ_{Nrela}$ and ERRQ are depicted in Figure 10 as the functions of N and σ. Figure 11 illustrates the dependence of the index MERR on N and σ.

The mean integral error ERRQ for N≥12,000 does not depend essentially on the number of measurements, either for small or large noises (see Figure 10b), while both the empirical index $ERRQ_{Nrel}$ and mean relative error MERR decrease exponentially with the increasing number of measurements, the MERR for N≥7000. For N≥1000, the MERR index does not exceed 1.01%, for N≥7000 it does not exceed 0.22%, while for N≥10,000, it falls below 0.05% even for the strongest disturbances. The globally optimal parameter $g^{*}$ was determined. As is seen from Figure 9, the algorithm practically ensures an excellent quality of the relaxation modulus approximation even for the strongest noises. The values of the relative relaxation modulus approximation error $ERRQ_{Nrel}$, already for the N≥100 measurements, do not exceed 3.3 × 10^−4^% and for N≥1000, fall below 8.3 × 10^−6^%. From the course of the mean integral sampling points-independent index ERRQ (40), as the function of N, we can conclude that the model is practically independent on the sampling instants for N≥12,000, independently on the measurement noises. The above combined with the close to zero values of $ERRQ_{Nrel}$ means the determining of the globally optimal model with the parameter $g^{*}$. In conclusion, the courses of both the index $ERRQ_{Nrel}$ (38), and the indices MERR (41) and ERRQ (40) as the functions of N, indicate the asymptotic independence of the model from the sampling points for a sufficiently large number of measurements.

## 4. Conclusions

The fractional Maxwell model allows for the modeling of a very wide range of stress relaxation processes in materials. The goal of the FMM identification is, generally, not to achieve a true description of the genuine relaxation modulus, but one that is “optimally accurate” in the assumed sense of the square weighted approximation error and does not depend on the particular sampling instants used in the stress relaxation experiment. The stochastic-type analytical analysis and numerical studies demonstrated that, despite the fact that the real description of the relaxation modulus is completely unknown, an arbitrarily exact approximation of the sampling points-independent optimal FMM can be identified based on the relaxation modulus data sampled randomly, according to respective randomization, when the number of the measurements applied in the experiment appropriately grow large. The four parameters of the approximate FMM are strongly consistent estimates of the parameters of the sampling points-independent model minimizing the integral square approximation error. The resulting identification scheme is simple and useful in application. It requires only the a priori, before the experiment is performed, independent random choice of the time instants at which the relaxation modulus is recorded from the assumed set according to a stationary rule.

Although this article is about modeling the relaxation modulus, the proposed identification scheme can also be successfully applied to the identification of the fractional-order models of creep compliance using the measurements obtained in the retardation test, whenever the respective set of sampling instants is open to manipulation during experimental data collecting. Therefore, the applicability of the identification asymptotically independent of the time instants used in the rheological experiment, to other fractional-order models determination, in particular, Kelvin–Voight, Zener and anti-Zener models, can be the subject of future research.

## Figures and Tables

**Table 1:** The components $\alpha^{*}$, $\beta^{*}$, $G_{e}^{*}$ and $\tau_{r}^{*}$ of the FMM parameter $g^{*}$ solving the optimal identification problem (27) and the optimal integral quadratic indices $Q\left(g^{*}\right)$ defined by (27) for the “real” relaxation modulus $G\left(t\right)$ (36).

$Q\left(g^{*}\right)\;\left[{kPa}^{2}\right]$	$\alpha^{*}\left[-\right]$	$\beta^{*}\left[-\right]$	$G_{e}^{*}\;\left[kPa\right]$	$\tau_{r}^{*}\left[s\right]$
5.2054279 × 10^−4^	0.920029	1.469033 × 10^−2^	3.086723	12.949456

**Table 2:** The elements $\alpha^{*}$, $\beta^{*}$, $G_{e}^{*}$ and $\tau_{r}^{*}$ of the FMM parameter $g^{*}$ solving the optimal identification task (27) and the optimal integral quadratic indices $Q\left(g^{*}\right)$ defined by (27) for the “real” relaxation modulus $G\left(t\right)$ (42), (43).

$Q\left(g^{*}\right)\;\left[{MPa}^{2}\right]$	$\alpha^{*}\left[-\right]$	$\beta^{*}\left[-\right]$	$G_{e}^{*}\;\left[MPa\right]$	$\tau_{r}^{*}\left[s\right]$
2.383349 × 10^−5^	0.736706	8.088257 × 10^−2^	1.2634125	6.397636 × 10^3^

**Figure A1:**
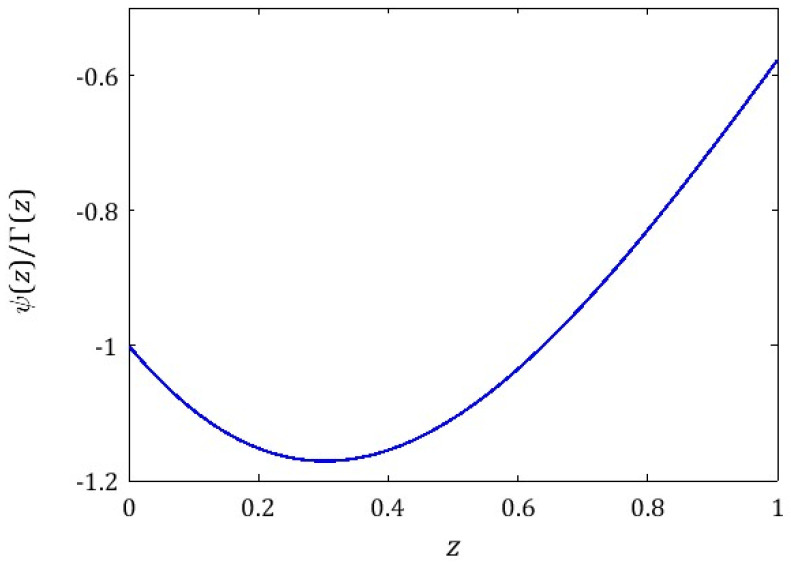
The quotient $\frac{\psi \left(z\right)}{\Gamma \left(z\right)}$ of the psi function $\psi \left(z\right)$ (A55) by the gamma function $\Gamma \left(z\right)$ for real argument 0≤z≤1.

## Data Availability

Data are contained within the article.

## Appendix A

### Appendix A.1. Proof of Theorem 1

Note, firstly, that the assumption $g\in G_{1}$ means, in particular, that $0<\beta_{0}\leq \;\beta <\alpha \leq 1$. The assumption t∈T, by definition of the set $T=\left[t_{0},T\right]$, where $t_{0}>0$, implies inequality t>0.

Differentiability and whence continuity of the relaxation modulus model $G_{M}\left(t,g\right)$ (7) with respect to parameter $G_{e}$ is obvious. The two-parameter Mitteg-Leffler function $E_{\kappa ,\mu }\left(x\right)$ (6) is known to be differentiable and continuous with respect to real argument x and parameters κ and μ [36]. Therefore, by (13) and (14), the differentiability of the function $G_{M}\left(t,g\right)$ with respect to the positive relaxation time parameter $\tau_{r}$ and parameters α and β directly results. To show that the condition (22) is satisfied, it is enough to prove that the partial derivatives of $G_{M}\left(t,g\right)$ with respect to the four model parameters are bounded uniformly on $T\times G_{1}$.

To prove the boundness of the partial derivatives with respect to the relaxation time parameter $\tau_{r}$ and the orders α and β of the stress and strain derivatives, the spectral representation (21) of the FMM will be applied together with the property concerning the absolute boundness, uniform on the set $T\times G_{1}$, of some definite integrals, which results from the following known [62] property concerning the absolute integrability of the product of absolutely integrable and bounded functions.

**Property** **A1** ([62])**.** *If the function* $f\left(x\right)$ *is absolutely integrable in the interval* $\left[a,\left.\infty \right)\right.$ *and the function* $g\left(x\right)$ *is bounded in* $\left[a,\left.\infty \right)\right.$*, then the product* $f\left(x\right)g\left(x\right)$ *is absolutely integrable in* $\left[a,\left.\infty \right)\right.$.

Let us consider definite integral

(A1) $$I_{0}\left(t,g\right)=\int_{0}^{\infty }r_{0}\left(v,t,g\right)f_{0}\left(v,t,g\right)dv,$$

where the function $f_{0}\left(v,t,g\right)$: $R_{+}\times T\times G_{0}\to R$ is absolutely integrable with respect to the variable of integration v in $R_{+}$, uniformly on the set $T\times G_{0}$, where $R_{+}=\left[0,\right.\left.\infty \right)$, $G_{0}\subset R^{k}$, k≥1 and the function $r_{0}\left(v,t,g\right):R_{+}\times T\times G_{0}\to R$ is absolutely bounded, uniformly on $R_{+}\times T\times G_{0}$. The first assumption means that there exists a positive constant $\bar{m}$ such that

(A2) $$\int_{0}^{\infty }\left|f_{0}\left(v,t,g\right)\right|dv\leq \bar{m}<\infty ,$$

for any t∈T and any $g\in G_{0}$, while the second assumption yields

(A3) $$\left|r_{0}\left(v,t,g\right)\right|\leq \overset{̿}{m}<\infty ,$$

for any $\left(v,t,g\right)\in R_{+}\times T\times G_{0}$. From Property A1, the convergence of the integral

(A4) $$\int_{0}^{\infty }\left|r_{0}\left(v,t,g\right)f_{0}\left(v,t,g\right)\right|dv,$$

and, in consequence, of the integral $I_{0}\left(t,g\right)$ (A1) for any t∈T and any $g\in G_{0}$ follows. In view of (A3) and (A4), we have

$$\left|I_{0}\left(t,g\right)\right|\leq \int_{0}^{\infty }\left|r_{0}\left(v,t,g\right)f_{0}\left(v,t,g\right)\right|dv\leq \overset{̿}{m}\int_{0}^{\infty }\left|f_{0}\left(v,t,g\right)\right|dv\leq \overset{̿}{m}\bar{m}=\bar{M}.$$

Therefore, the next result holds.

**Property** **A2.** *If the function* $f_{0}\left(v,t,g\right)$ *is absolutely integrable with respect to* v *in the interval* $R_{+}=\left[0,\left.\infty \right)\right.$*, uniformly on the set* $T\times G_{0}$ *of the rest arguments* $\left(t,g\right)$*, and the function* $r_{0}\left(v,t,g\right)$ *is bounded uniformly on* $R_{+}\times T\times G_{0}$*, then the product* $r_{0}\left(v,t,g\right)f_{0}\left(v,t,g\right)$ *is absolutely integrable in* $R_{+}$ *for any* $\left(t,g\right)\in T\times G_{0}$ *and the integral* $I_{0}\left(t,g\right)$ *(A1) is absolutely bounded uniformly on* $T\times G_{0}$.

Below, the proof is divided into four parts related to the four model parameters.

#### Appendix A.1.1. Uniform on $T\times G_{1}$ Boundness of the FMM Derivative with Respect to $G_{e}$

From (7), we have

(A5) $$\frac{\partial G_{M}\left(t,g\right)}{\partial G_{e}}={\left(\frac{t}{\tau_{r}}\right)}^{-\beta }E_{\alpha -\beta ,1-\beta }\left(-{\left(\frac{t}{\tau_{r}}\right)}^{\alpha -\beta }\right),$$

whence, by (12) and (16), the uniform boundness of the above derivative on the set $T\times G_{1}$ follows.

#### Appendix A.1.2. Uniform on $T\times G_{1}$ Boundness of the FMM Derivative with Respect to $\tau_{r}$

By (7) and (13), we can express $G_{M}\left(t,g\right)$ as follows

$$G_{M}\left(t,g\right)=G_{e}e_{\alpha -\beta ,1-\beta }\left(\frac{t}{\tau_{r}};1\right).$$

Whence, the partial derivative with respect to the relaxation time, in the respective notation, is given by

(A6) $$\frac{\partial G_{M}\left(t,g\right)}{\partial \tau_{r}}=G_{e}\frac{\left(-1\right)t}{{\left(\tau_{r}\right)}^{2}}{\left.\frac{d}{dx}e_{\alpha -\beta ,1-\beta }\left(x;1\right)\right|}_{x=\frac{t}{\tau_{r}}},$$

where, due to the complete monotonicity of the function $e_{\alpha -\beta ,1-\beta }\left(x;1\right)$, the negative derivative $\frac{d}{dx}e_{\alpha -\beta ,1-\beta }\left(x;1\right)$ monotonically increases to zero for x>0.

From (A5) and (A6), the nonnegative definiteness of $\frac{\partial G_{M}\left(t,g\right)}{\partial G_{e}}$ and $\frac{\partial G_{M}\left(t,g\right)}{\partial \tau_{r}}$, being positive for any $g\in G_{1}$ and t<∞, follows; therefore, Property 1 is formulated.

To examine the asymptotic properties of $\frac{\partial G_{M}\left(t,g\right)}{\partial \tau_{r}}$ as t→∞ and as $t\to t_{0}$, let us express (A6), applying the known differentiation formula [4] (Equation (E.55))

$$e_{\kappa ,\mu }\left(x;\lambda \right)=\frac{d}{dx}e_{\kappa ,\mu +1}\left(x;\lambda \right),$$

in the form

$$\frac{\partial G_{M}\left(t,g\right)}{\partial \tau_{r}}=G_{e}\frac{\left(-1\right)t}{{\left(\tau_{r}\right)}^{2}}e_{\alpha -\beta ,-\beta }\left(\frac{t}{\tau_{r}};1\right),$$

or, having in mind definition (13), directly in terms of the Mittag-Leffler function

(A7) $$\frac{\partial G_{M}\left(t,g\right)}{\partial \tau_{r}}=\left(-1\right)\frac{G_{e}}{\tau_{r}}{\left(\frac{t}{\tau_{r}}\right)}^{-\beta }E_{\alpha -\beta ,-\beta }\left(-{\left(\frac{t}{\tau_{r}}\right)}^{\alpha -\beta }\right).$$

The following asymptotic approximation [63] (Equation (12)), see also [4] (Equation (E.30)):

$$E_{\kappa ,\mu }\left(-t^{\kappa }\right)≅\sum_{n=1}^{\infty }{\left(-1\right)}^{n-1}\frac{t^{-\kappa n}}{\Gamma \left(\mu -\kappa n\right)},$$

which holds for t→∞, applied to (A7), yields

$$\frac{\partial G_{M}\left(t,g\right)}{\partial \tau_{r}}≅\left(-1\right)\frac{G_{e}}{\tau_{r}}{\left(\frac{t}{\tau_{r}}\right)}^{-\beta }\left[\frac{{\left(\frac{t}{\tau_{r}}\right)}^{-\left(\alpha -\beta \right)}}{\Gamma \left(-\alpha \right)}+\sum_{n=2}^{\infty }{\left(-1\right)}^{n-1}\frac{{\left(\frac{t}{\tau_{r}}\right)}^{-\left(\alpha -\beta \right)n}}{\Gamma \left[-\beta -\left(\alpha -\beta \right)n\right]}\right],$$

whence, for large times, especially for ${\left(\frac{t}{\tau_{r}}\right)}^{\alpha -\beta }\gg 1$, we obtain the asymptotic long-time approximation

$$\frac{\partial G_{M}\left(t,g\right)}{\partial \tau_{r}}≅\left(-1\right)\frac{G_{e}}{\tau_{r}\Gamma \left(-\alpha \right)}{\left(\frac{t}{\tau_{r}}\right)}^{-\alpha },$$

where ≅ means “approximately equal”. Therefore, derivative $\frac{\partial G_{M}\left(t,g\right)}{\partial \tau_{r}}$ tends to zero as t→∞ for any admissible parameter ${g\in G}_{1}$.

To estimate the value of $\frac{\partial G_{M}\left(t,g\right)}{\partial \tau_{r}}$ (A7) for $t=t_{0}$, the series representation

$$\frac{\partial G_{M}\left(t,g\right)}{\partial \tau_{r}}=\frac{G_{e}}{\tau_{r}}{\left(\frac{t}{\tau_{r}}\right)}^{-\beta }\sum_{n=0}^{\infty }\frac{{\left(-1\right)}^{n+1}{\left(\frac{t}{\tau_{r}}\right)}^{\left(\alpha -\beta \right)n}}{\Gamma \left[\left(\alpha -\beta \right)n-\beta \right]}$$

resulting directly from (6) and (A7), is used. The first summand of the series is positive, while the next elements are positive or negative, depending on the index n and the relation between parameters α and β. Since

(A8) $$\left|{\left(\frac{t}{\tau_{r}}\right)}^{-\beta }\sum_{n=0}^{\infty }\frac{{\left(-1\right)}^{n+1}{\left(\frac{t}{\tau_{r}}\right)}^{\left(\alpha -\beta \right)n}}{\Gamma \left[\left(\alpha -\beta \right)n-\beta \right]}\right|\leq {\left(\frac{t}{\tau_{r}}\right)}^{-\beta }\sum_{n=0}^{\infty }\frac{{\left(\frac{t}{\tau_{r}}\right)}^{\left(\alpha -\beta \right)n}}{\left|\Gamma \left[\left(\alpha -\beta \right)n-\beta \right]\right|},$$

and the argument of the gamma function is such that

$$\left(\alpha -\beta \right)n-\beta \geq -\beta >-1,$$

which in view of the monotonicity of the gamma function implies

(A9) $$\left|\Gamma \left[\left(\alpha -\beta \right)n-\beta \right]\right|\geq \Gamma \left[x_{min}\right]=0.8856032>0.8856,$$

where $x_{min}≅1.4616321$ is the real nonnegative argument at which a minimum of the function $\Gamma \left(x\right)$ occurs [64]. In view of (A8) and (A9), having in mind the nonnegative definiteness of $\frac{\partial G_{M}\left(t,g\right)}{\partial \tau_{r}}$, we obtain the following estimation

$$\frac{\partial G_{M}\left(t,g\right)}{\partial \tau_{r}}<\frac{G_{e}}{{1.1292\tau }_{r}}{\left(\frac{t}{\tau_{r}}\right)}^{-\beta }\sum_{n=0}^{\infty }{\left(\frac{t}{\tau_{r}}\right)}^{\left(\alpha -\beta \right)n},$$

which, by the inequalities $\tau_{rmin}\leq \tau_{r}\leq \tau_{rmax}$, for $t=t_{0}$, implies the next estimation

$${\left.\frac{\partial G_{M}\left(t,g\right)}{\partial \tau_{r}}\right|}_{t=t_{0}}<\frac{G_{e}}{1.1292\tau_{rmin}}{\left(\frac{\tau_{rmax}}{\tau_{rmin}}\right)}^{\beta }{\left(\frac{t_{0}}{\tau_{rmin}}\right)}^{-\beta }\sum_{n=0}^{\infty }{\left(\frac{t_{0}}{\tau_{rmin}}\right)}^{\left(\alpha -\beta \right)n}.$$

By the assumption $t_{0}<\tau_{rmin}$, the above estimation can be rewritten in compact form as

(A10) $${\left.\frac{\partial G_{M}\left(t,g\right)}{\partial \tau_{r}}\right|}_{t=t_{0}}<\frac{G_{e}}{1.1292\tau_{rmin}}{\left(\frac{\tau_{rmax}}{\tau_{rmin}}\right)}^{\beta }\frac{{\left(\frac{t_{0}}{\tau_{rmin}}\right)}^{-\beta }}{1-{\left(\frac{t_{0}}{\tau_{rmin}}\right)}^{\alpha -\beta }}.$$

Since, for an arbitrary $\beta_{0}\leq \;\beta <\alpha \leq 1$, we have

$$\frac{{\left(\frac{t_{0}}{\tau_{rmin}}\right)}^{-\beta }}{1-{\left(\frac{t_{0}}{\tau_{rmin}}\right)}^{\alpha -\beta }}=\frac{1}{{\left(\frac{t_{0}}{\tau_{rmin}}\right)}^{\beta }-{\left(\frac{t_{0}}{\tau_{rmin}}\right)}^{\alpha }}<\frac{1}{{\left(\frac{t_{0}}{\tau_{rmin}}\right)}^{\beta }},$$

inequality (A10) for any $g\in G_{1}$ implies

$${\left.\frac{\partial G_{M}\left(t,g\right)}{\partial \tau_{r}}\right|}_{t=t_{0}}<\frac{G_{e}}{1.1292\tau_{rmin}}{\left(\frac{\tau_{rmax}}{t_{0}}\right)}^{\beta },$$

which means that

$${\left.\frac{\partial G_{M}\left(t,g\right)}{\partial \tau_{r}}\right|}_{t=t_{0}}<\frac{G_{emax}\;}{1.1292\tau_{rmin}}\frac{\tau_{rmax}}{t_{0}}=\frac{G_{emax}\;}{1.1292\tau_{rmin}}m_{0},$$

i.e., the derivative for $t=t_{0}$ is bounded, uniformly on the set $G_{1}$, where positive parameter $m_{0}$ is defined in Equation (16).

Since the continuous function $\frac{\partial G_{M}\left(t,g\right)}{\partial \tau_{r}}$ of the time t is bounded for $t=t_{0}$ and for t→∞ for any fixed $g\in G_{1}$, derivative $\frac{\partial G_{M}\left(t,g\right)}{\partial \tau_{r}}$ as a function of the time is bounded both for the bounded and not-bounded set T. However, due to the non-compactness of the set $G_{1}$, from which α=β is excluded, the uniform on $T\times G_{1}$ boundness of $\frac{\partial G_{M}\left(t,g\right)}{\partial \tau_{r}}$ is not obvious. Therefore, it should be examined if the maximum of $\frac{\partial G_{M}\left(t,g\right)}{\partial \tau_{r}}$ (with respect to the time) is bounded, as $\alpha \to \beta^{+}$. To this end, an alternative to (A6) and (A7), the representation of $\frac{\partial G_{M}\left(t,g\right)}{\partial \tau_{r}}$ is derived based on the spectral representation given by Equation (21).

Differentiation of (21) on both sides with respect to $\tau_{r}$ yields

(A11) $$\frac{\partial G_{M}\left(t,g\right)}{\partial \tau_{r}}=G_{e}\alpha \frac{\tau_{r}^{\alpha -1}}{\pi }\int_{0}^{\infty }r\left(v,g\right)v^{\alpha -1}e^{-tv}dv+G_{e}\frac{\tau_{r}^{\alpha }}{\pi }\int_{0}^{\infty }\frac{\left(\alpha -\beta \right){\left(\tau_{r}v\right)}^{\alpha -\beta -1}\sin\left(\pi \beta \right)}{{\left(\tau_{r}v\right)}^{2\left(\alpha -\beta \right)}+2{\left(\tau_{r}v\right)}^{\alpha -\beta }\cos\left[\pi \left(\alpha -\beta \right)\right]+1}v^{\alpha }e^{-tv}dv-G_{e}\frac{\tau_{r}^{\alpha }}{\pi }\int_{0}^{\infty }\frac{2\left(\alpha -\beta \right){\left(\tau_{r}v\right)}^{2\left(\alpha -\beta \right)-1}+2{\left(\alpha -\beta \right)\left(\tau_{r}v\right)}^{\alpha -\beta -1}\cos\left[\pi \left(\alpha -\beta \right)\right]}{{\left(\tau_{r}v\right)}^{2\left(\alpha -\beta \right)}+2{\left(\tau_{r}v\right)}^{\alpha -\beta }\cos\left[\pi \left(\alpha -\beta \right)\right]+1}r\left(v,g\right)v^{\alpha }e^{-tv}dv,$$

where the function

(A12) $$r\left(v,g\right)=\frac{{\left(\tau_{r}v\right)}^{\alpha -\beta }\sin\left(\pi \beta \right)+\sin\left(\pi \alpha \right)}{{\left(\tau_{r}v\right)}^{2\left(\alpha -\beta \right)}+2{\left(\tau_{r}v\right)}^{\alpha -\beta }\cos\left[\pi \left(\alpha -\beta \right)\right]+1},$$

by (21), is such that

(A13) $$G_{M}\left(t,g\right)=G_{e}\frac{\tau_{r}^{\alpha }}{\pi }\int_{0}^{\infty }r\left(v,g\right)v^{\alpha -1}e^{-tv}dv.$$

Whence, introducing the notations

(A14) $$r_{1}\left(v,g\right)=\frac{{\left(\tau_{r}v\right)}^{\alpha -\beta }\sin\left(\pi \beta \right)}{{\left(\tau_{r}v\right)}^{2\left(\alpha -\beta \right)}+2{\left(\tau_{r}v\right)}^{\alpha -\beta }\cos\left[\pi \left(\alpha -\beta \right)\right]+1},$$

(A15) $$r_{2}\left(v,g\right)=\frac{{\left(\tau_{r}v\right)}^{2\left(\alpha -\beta \right)}+{\left(\tau_{r}v\right)}^{\alpha -\beta }\cos\left[\pi \left(\alpha -\beta \right)\right]}{{\left(\tau_{r}v\right)}^{2\left(\alpha -\beta \right)}+2{\left(\tau_{r}v\right)}^{\alpha -\beta }\cos\left[\pi \left(\alpha -\beta \right)\right]+1},$$

Equation (A11) can be rewritten as

$$\frac{\partial G_{M}\left(t,g\right)}{\partial \tau_{r}}=\frac{\alpha }{\tau_{r}}G_{M}\left(t,g\right)+\left(\alpha -\beta \right)G_{e}\frac{\tau_{r}^{\alpha -1}}{\pi }\int_{0}^{\infty }r_{1}\left(v,g\right)v^{\alpha -1}e^{-tv}dv-\;2\left(\alpha -\beta \right)G_{e}\frac{\tau_{r}^{\alpha -1}}{\pi }\int_{0}^{\infty }r_{2}\left(v,g\right)r\left(v,g\right)v^{\alpha -1}e^{-tv}dv,$$

or in a more compact form as a linear combination:

(A16) $$\frac{\partial G_{M}\left(t,g\right)}{\partial \tau_{r}}=\frac{\alpha }{\tau_{r}}G_{M}\left(t,g\right)+\left(\alpha -\beta \right)G_{e}\frac{\tau_{r}^{\alpha -1}}{\pi }I_{1}\left(t,g\right)-2\left(\alpha -\beta \right)G_{e}\frac{\tau_{r}^{\alpha -1}}{\pi }I_{2}\left(t,g\right),$$

where the integrals:

(A17) $$I_{1}\left(t,g\right)=\int_{0}^{\infty }r_{1}\left(v,g\right)v^{\alpha -1}e^{-tv}dv,$$

(A18) $$I_{2}\left(t,g\right)=\int_{0}^{\infty }\;r_{2}\left(v,g\right)r\left(v,g\right)v^{\alpha -1}e^{-tv}dv$$

The denominator

(A19) $$q\left(v,g\right)={\left(\tau_{r}v\right)}^{2\left(\alpha -\beta \right)}+2{\left(\tau_{r}v\right)}^{\alpha -\beta }\cos\left[\pi \left(\alpha -\beta \right)\right]+1$$

of the fractions $r\left(v,g\right)$, $r_{1}\left(v,g\right)$ and $r_{2}\left(v,g\right)$ is positive for any v≥0, whenever α−β≠1, i.e., for any admissible parameter $g\in G_{1}$, which satisfies the following inequalities

$$0<\alpha -\beta \leq 1-\beta \leq 1-\beta_{0}<1.$$

For $\alpha \to \beta^{+}$, the denominator $q\left(v,g\right)\to {\left[{\left(\tau_{r}v\right)}^{\alpha -\beta }+1\right]}^{2}\geq 1$. By (A13), (17) and nonnegative definiteness of $r\left(v,g\right)$ (A12) on the set $R_{+}\times G_{1}$, we have

$$\int_{0}^{\infty }\left|r\left(v,g\right)v^{\alpha -1}e^{-tv}\right|dv=\int_{0}^{\infty }r\left(v,g\right)v^{\alpha -1}e^{-tv}dv=\frac{\pi }{G_{e}\tau_{r}^{\alpha }}G_{M}\left(t,g\right)\leq \frac{\pi }{G_{e}\tau_{r}^{\alpha }}M_{1}$$

for any $\left(t,g\right)\in T\times G_{1}$, i.e., the function $r\left(v,g\right)v^{\alpha -1}e^{-tv}$ as the function of the variable v is absolutely integrable, uniformly on the set $T\times G_{1}$, with the constant $\bar{m}$ (compare (A2)) given by

(A20) $$\int_{0}^{\infty }\left|r\left(v,g\right)v^{\alpha -1}e^{-tv}\right|dv\leq \bar{m}=\frac{\pi }{G_{emin}\gamma_{2}}M_{1},$$

where the parameter

(A21) $$0<\gamma_{2}=\underset{\beta_{0}\leq \alpha \leq 1}{\min}\tau_{rmin}^{\alpha }<\infty .$$

In view of Property A2, bearing in mind inequality (17), or (15), to prove the absolute uniform boundness of the derivative $\frac{\partial G_{M}\left(t,g\right)}{\partial \tau_{r}}$ (A16) on the set $T\times G_{1}$, it is enough to demonstrate that the integrals $I_{1}\left(t,g\right)$ (A17) and $I_{2}\left(t,g\right)$ (A18) are convergent and absolutely bounded, uniformly on the set $T\times G_{1}$. For this purpose, we express the two integrals as definite integrals of the product of some absolutely integrable function and a bounded function.

The continuous, nonnegative definite for any $\left(v,g\right)\in R_{+}\times G_{1}$, function $r_{1}\left(v,g\right)$ (A14) is equal to zero for v=0, tends to zero for v→∞ and takes the maximal value for $v=1/\tau_{r}$, independently on the values of α and β. Whence, for any $\left(v,g\right)\in R_{+}\times G_{1}$, the inequalities hold

(A22) $$\left|r_{1}\left(v,g\right)\right|\leq \frac{\sin\left(\pi \beta \right)}{2\cos\left[\pi \left(\alpha -\beta \right)\right]+2}\leq \frac{1}{2\cos\left[\pi \left(1-\beta_{0}\right)\right]+2}=\frac{1}{2-2\cos\left(\pi \beta_{0}\right)}=m_{1}<\infty ,$$

i.e., $r_{1}\left(v,g\right)$ is absolutely bounded uniformly on $R_{+}\times G_{1}$. By the following notable integral [4] (Equation (A.21):

(A23) $$\int_{0}^{\infty }v^{\alpha -1}e^{-tv}dv=\int_{0}^{\infty }\left|v^{\alpha -1}e^{-tv}\right|dv=\frac{\Gamma \left(\alpha \right)}{t^{\alpha }},$$

which holds for any α>0 and t>0, $v^{\alpha -1}e^{-tv}$ is the absolutely integrable function of the variable v≥0 for any t∈T. Recalling the definitions of the sets T and $G_{1}$ (19), and the monotonicity of the gamma function $\Gamma \left(\alpha \right)$ for 0<α≤1, we immediately obtain the estimation

(A24) $$\int_{0}^{\infty }v^{\alpha -1}e^{-tv}dv\leq \frac{\Gamma \left(\beta_{0}\right)}{\gamma_{1}},$$

valid for any t∈T and any $g\in G_{1}$, where

(A25) $$0<\gamma_{1}=\underset{\beta_{0}\leq \alpha \leq 1}{\min}t_{0}^{\alpha }<\infty .$$

Therefore, according to Property A2, the integral $I_{1}\left(t,g\right)$ (A17) is convergent for any t>0 and any $g\in G_{1}$ and absolutely bounded by the upper bound equal to $m_{1}\Gamma \left(\beta_{0}\right)/\gamma_{1}$, uniformly on the set $T\times G_{1}$.

It is easy to check that continuous function $r_{2}\left(v,g\right)$ (A15) is equal to zero for v=0 and tends to 1, as v→∞, independently on the values of α and β from the set $G_{1}$, i.e., for α>β. Function $r_{2}\left(v,g\right)$ can be expressed as

$$r_{2}\left(v,g\right)=1-\frac{{\left(\tau_{r}v\right)}^{\alpha -\beta }\cos\left[\pi \left(\alpha -\beta \right)\right]}{q\left(v,g\right)}-\frac{1}{q\left(v,g\right)},$$

with $q\left(v,g\right)$ defined by (A19), where the absolute value of the second summand takes the maximal value for $v=1/\tau_{r}$, while the third summand takes the maximal value whenever ${\left(\tau_{r}v\right)}^{\alpha -\beta }=-\cos\left[\pi \left(\alpha -\beta \right)\right]$, if $\cos\left[\pi \left(\alpha -\beta \right)\right]<0$, and for v=0 in the opposite case. Therefore, for any $\left(v,g\right)\in R_{+}\times G_{1}$, the next estimation holds

(A26) $$\left|r_{2}\left(v,g\right)\right|\leq 1+\frac{\left|\cos\left[\pi \left(\alpha -\beta \right)\right]\right|}{2\cos\left[\pi \left(\alpha -\beta \right)\right]+2}+m\left(\alpha ,\beta \right),$$

where

(A27) $$m\left(\alpha ,\beta \right)=\left\{\begin{array}{l} \frac{1}{1-\cos^{2}\left[\pi \left(\alpha -\beta \right)\right]}\;\;\;\;\;\;if\;\cos\left[\pi \left(\alpha -\beta \right)\right]<0\;\\ 1\;\;\;\;\;\;\;\;\;\;\;\;\;\;\;\;\;\;\;\;\;\;\;\;\;\;\;if\;\cos\left[\pi \left(\alpha -\beta \right)\right]\geq 0 \end{array}\right..$$

Since, for $g\in G_{1}$, the inequality α>β holds, for $\alpha \to \beta^{+}$, we have $\cos\left[\pi \left(\alpha -\beta \right)\right]\to 1^{-}$ and $m\left(\alpha ,\beta \right)\to 1$. Simultaneously, if α→1 and $\beta \to \beta_{0}$, then $m\left(\alpha ,\beta \right)\to \frac{1}{1-\cos^{2}\left(\pi \beta_{0}\right)}>1$, whenever $\beta_{0}<\frac{1}{2}$ and $m\left(\alpha ,\beta \right)\to 1$ for $\beta_{0}\geq \frac{1}{2}$. Therefore, for any $\left(v,g\right)\in R_{+}\times G_{1}$, by (A26) and (A27), we have

(A28) $$\left|r_{2}\left(v,g\right)\right|\leq 1+\frac{1}{2-2\cos\left(\pi \beta_{0}\right)}+\frac{1}{1-\cos^{2}\left(\pi \beta_{0}\right)}=1+m_{1}+m_{2}<\infty ,$$

where

(A29) $$m_{2}=\frac{1}{1-\cos^{2}\left(\pi \beta_{0}\right)}<\infty ,$$

and according to Property A2, the integral $I_{2}\left(t,g\right)$ (A18) is convergent for any t∈T and any $g\in G_{1}$ and absolutely bounded uniformly on $T\times G_{1}$, with the upper bound $\frac{\pi \left(1+m_{1}+m_{2}\right)}{G_{emin}\gamma_{2}}M_{1}$ resulting from (A20) and (A28).

Combining the absolute boundness of the three summands of the right-hand side of (A16), uniform on the set $T\times G_{1}$, the respective uniform boundness of $\frac{\partial G_{M}\left(t,g\right)}{\partial \tau_{r}}$ is proved.

#### Appendix A.1.3. Uniform on $T\times G_{1}$ Boundness of the FMM Derivative with Respect to β

Differentiation of Equation (21) on both sides with respect to β yields

$$\frac{\partial G_{M}\left(t,g\right)}{\partial \beta }=G_{e}\frac{\tau_{r}^{\alpha }}{\pi }\int_{0}^{\infty }\frac{-ln\left(\tau_{r}v\right){\left(\tau_{r}v\right)}^{\alpha -\beta }\sin\left(\pi \beta \right)+\pi {\left(\tau_{r}v\right)}^{\alpha -\beta }\cos\left(\pi \beta \right)}{{\left(\tau_{r}v\right)}^{2\left(\alpha -\beta \right)}+2{\left(\tau_{r}v\right)}^{\alpha -\beta }\cos\left[\pi \left(\alpha -\beta \right)\right]+1}v^{\alpha -1}e^{-tv}dv+2G_{e}\frac{\tau_{r}^{\alpha }}{\pi }\int_{0}^{\infty }r\left(v,g\right)\frac{ln\left(\tau_{r}v\right){\left(\tau_{r}v\right)}^{2\left(\alpha -\beta \right)}+ln\left(\tau_{r}v\right){\left(\tau_{r}v\right)}^{\alpha -\beta }\cos\left[\pi \left(\alpha -\beta \right)\right]-\pi {\left(\tau_{r}v\right)}^{\alpha -\beta }sin\left[\pi \left(\alpha -\beta \right)\right]}{{\left(\tau_{r}v\right)}^{2\left(\alpha -\beta \right)}+2{\left(\tau_{r}v\right)}^{\alpha -\beta }\cos\left[\pi \left(\alpha -\beta \right)\right]+1}v^{\alpha -1}e^{-tv}dv$$

where $r\left(v,g\right)$ is given by (A12), which using the notations $r_{1}\left(v,g\right)$ (A14) and $r_{2}\left(v,g\right)$ (A15) and introducing functions

(A30) $$r_{3}\left(v,g\right)=\frac{{\left(\tau_{r}v\right)}^{\alpha -\beta }\cos\left(\pi \beta \right)}{{\left(\tau_{r}v\right)}^{2\left(\alpha -\beta \right)}+2{\left(\tau_{r}v\right)}^{\alpha -\beta }\cos\left[\pi \left(\alpha -\beta \right)\right]+1},$$

(A31) $$r_{4}\left(v,g\right)=\frac{{\left(\tau_{r}v\right)}^{\alpha -\beta }sin\left[\pi \left(\alpha -\beta \right)\right]}{{\left(\tau_{r}v\right)}^{2\left(\alpha -\beta \right)}+2{\left(\tau_{r}v\right)}^{\alpha -\beta }\cos\left[\pi \left(\alpha -\beta \right)\right]+1},$$

can be rewritten in compact form as a linear combination:

(A32) $$\frac{\partial G_{M}\left(t,g\right)}{\partial \beta }=G_{e}\tau_{r}^{\alpha }I_{3}\left(t,g\right)-2G_{e}\tau_{r}^{\alpha }I_{4}\left(t,g\right)-G_{e}\frac{\tau_{r}^{\alpha }}{\pi }I_{5}\left(t,g\right)+2G_{e}\frac{\tau_{r}^{\alpha }}{\pi }I_{6}\left(t,g\right),$$

of four integrals:

(A33) $$I_{3}\left(t,g\right)=\int_{0}^{\infty }r_{3}\left(v,g\right)v^{\alpha -1}e^{-tv}dv,$$

(A34) $$I_{4}\left(t,g\right)=\int_{0}^{\infty }\;r_{4}\left(v,g\right)r\left(v,g\right)v^{\alpha -1}e^{-tv}dv,$$

(A35) $$I_{5}\left(t,g\right)=\int_{0}^{\infty }r_{1}\left(v,g\right)ln\left(\tau_{r}v\right)v^{\alpha -1}e^{-tv}dv,$$

(A36) $$I_{6}\left(t,g\right)=\int_{0}^{\infty }r_{2}\left(v,g\right)r\left(v,g\right)ln\left(\tau_{r}v\right)v^{\alpha -1}e^{-tv}dv.$$

To prove the absolute boundness of the derivative $\frac{\partial G_{M}\left(t,g\right)}{\partial \beta }$ (A32), uniform on the set $T\times G_{1}$, it is enough to demonstrate that the four above integrals are convergent and absolutely bounded, uniformly on $T\times G_{1}$.

For any $\left(v,g\right)\in R_{+}\times G_{1}$, the continuous function $r_{3}\left(v,g\right)$ (A30) satisfies the following inequalities (compare (A14) and (A22))

(A37) $$\left|r_{3}\left(v,g\right)\right|\leq \frac{\left|\cos\left(\pi \beta \right)\right|}{2\cos\left[\pi \left(\alpha -\beta \right)\right]+2}\leq \frac{1}{2\cos\left[\pi \left(1-\beta_{0}\right)\right]+2}=\frac{1}{2-2\cos\left(\pi \beta_{0}\right)}=m_{1},$$

where $m_{1}$ is defined in (A22), i.e., $r_{3}\left(v,g\right)$ is absolutely bounded, uniformly on $R_{+}\times G_{1}$, which combined with the absolutely integrability of the function $v^{\alpha -1}e^{-tv}$ for any t∈T and any $g\in G_{1}$, according to Property A2, implies the convergence of the integral $I_{3}\left(t,g\right)$ (A33) for any t∈T and any $g\in G_{1}$ and its absolute boundness by the upper bound $m_{1}\Gamma \left(\beta_{0}\right)/\gamma_{1}$ resulting from and (A24), (A25) and (A37), valid uniformly on the set $T\times G_{1}$.

The nonnegative function $r_{4}\left(v,g\right)$ (A31) is for any $\left(v,g\right)\in R_{+}\times G_{1}$ bounded by

(A38) $$r_{4}\left(v,g\right)\leq \frac{sin\left[\pi \left(\alpha -\beta \right)\right]}{2\cos\left[\pi \left(\alpha -\beta \right)\right]+2}\leq \frac{1}{2\cos\left[\pi \left(1-\beta_{0}\right)\right]+2}=\frac{1}{2-2\cos\left(\pi \beta_{0}\right)}=m_{1},$$

similarly to $r_{1}\left(v,g\right)$ (A14) and $r_{3}\left(v,g\right)$ (A30), which combined with the absolutely integrability of $r\left(v,g\right)v^{\alpha -1}e^{-tv}$ for any $g\in G_{1}$ and any t∈T, implies the convergence of the integral $I_{4}\left(t,g\right)$ (A34) for any $\left(t,g\right)\in T\times G_{1}$ and the absolute boundness of $I_{4}\left(t,g\right)$ by the upper bound $\frac{\pi {\;m}_{1}}{G_{emin}\gamma_{2}}M_{1}$, derived from (A20) and (A38), uniformly on the set $T\times G_{1}$.

To demonstrate the convergence and uniform boundness of the integrals $I_{5}\left(t,g\right)$ (A35) and $I_{6}\left(t,g\right)$ (A36), let us express them as follows

(A39) $$I_{5}\left(t,g\right)=\int_{0}^{\infty }r_{12}\left(v,g\right)r_{5}\left(v,t,g\right)v^{\alpha -1}e^{-\frac{1}{2}tv}dv,$$

(A40) $$I_{6}\left(t,g\right)=\int_{0}^{\infty }r_{22}\left(v,g\right)r_{5}\left(v,t,g\right)r\left(v,g\right)v^{\alpha -1}e^{-\frac{1}{2}tv}dv.$$

where, by (A14) and (A15)

(A41) $$r_{12}\left(v,g\right)=\frac{\sin\left(\pi \beta \right)}{{\left(\tau_{r}v\right)}^{2\left(\alpha -\beta \right)}+2{\left(\tau_{r}v\right)}^{\alpha -\beta }\cos\left[\pi \left(\alpha -\beta \right)\right]+1},$$

(A42) $$r_{22}\left(v,g\right)=\frac{{\left(\tau_{r}v\right)}^{\alpha -\beta }+\cos\left[\pi \left(\alpha -\beta \right)\right]}{{\left(\tau_{r}v\right)}^{2\left(\alpha -\beta \right)}+2{\left(\tau_{r}v\right)}^{\alpha -\beta }\cos\left[\pi \left(\alpha -\beta \right)\right]+1},$$

and nonnegative continuous function

(A43) $$r_{5}\left(v,t,g\right)={\left(\tau_{r}v\right)}^{\alpha -\beta }ln\left(\tau_{r}v\right)e^{-\frac{1}{2}tv}=\frac{ln\left(\tau_{r}v\right){\left(\tau_{r}v\right)}^{\alpha -\beta }}{e^{\frac{1}{2}tv}}.$$

Since (compare (A13)),

$$G_{M}\left(\frac{t}{2},g\right)=G_{e}\frac{\tau_{r}^{\alpha }}{\pi }\int_{0}^{\infty }r\left(v,g\right)v^{\alpha -1}e^{-\frac{1}{2}tv}dv,$$

the nonnegative function $r\left(v,g\right)v^{\alpha -1}e^{-\frac{1}{2}tv}$ is absolutely integrable for any $\left(t,g\right)\in T\times G_{1}$ with the upper bound $\frac{\pi }{G_{emin}\gamma_{2}}M_{1}$; compare (A20). In turn, by (A23), $v^{\alpha -1}e^{-\frac{1}{2}tv}$ is an absolutely integrable function of the variable v≥0, uniformly on the set $T\times G_{1}$, with the upper bound $2\Gamma \left(\beta_{0}\right)/\gamma_{1}$; compare (A24) and (A25).

Functions $r_{12}\left(v,g\right)$ (A41) and $r_{22}\left(v,g\right)$ (A42) are absolutely bounded uniformly on $R_{+}\times G_{1}$, since the following estimations hold for any $\left(v,g\right)\in R_{+}\times G_{1}$:

(A44) $$r_{12}\left(v,g\right)\leq \frac{1}{1-\cos^{2}\left(\pi \beta_{0}\right)}=m_{2},$$

(A45) $$\left|r_{22}\left(v,g\right)\right|\leq \frac{1}{2-2\cos\left(\pi \beta_{0}\right)}+\frac{1}{1-\cos^{2}\left(\pi \beta_{0}\right)}=m_{1}+m_{2}<\infty ,$$

where constants $m_{1}$ and $m_{2}$ are defined in (A22) and (A29), respectively.

To examine the properties of $r_{5}\left(v,t,g\right)$ (A43), the asymptotic properties as $v\to 0^{+}$ and v→∞ are studied. This function is expressed as

$$r_{5}\left(v,t,g\right)=\frac{ln\left(\tau_{r}v\right)}{{\left(\tau_{r}v\right)}^{-\left(\alpha -\beta \right)}e^{\frac{1}{2}tv}},$$

where the nominator tends to −∞ and the denominator tends to +∞, as the variable $v\to 0^{+}$. Therefore, by applying the L’Hospital’s rule, in view of α>β>0, we obtain

$$\underset{v\to 0^{+}}{\lim}r_{5}\left(v,t,g\right)=\underset{v\to 0^{+}}{lim}\frac{1}{\left[-\tau_{r}\left(\alpha -\beta \right)+\frac{1}{2}t\;\tau_{r}v\right]{\left(\tau_{r}v\right)}^{-\left(\alpha -\beta \right)}e^{\frac{1}{2}tv}}=0^{-}.$$

Since ${\left(\tau_{r}v\right)}^{\alpha -\beta }ln\left(\tau_{r}v\right)$ tends to +∞, while $e^{-\frac{1}{2}tv}$ tends to zero, as the variable v tends to infinity, using the L’Hospital’s rule double times to the right expression in (A43), we have

$$\underset{v\to \infty }{\lim}r_{5}\left(v,t,g\right)=\underset{v\to \infty }{lim}\frac{\tau_{r}+\left(\alpha -\beta \right)ln\left(\tau_{r}v\right)\tau_{r}}{\frac{1}{2}te^{\frac{1}{2}tv}{\left(\tau_{r}v\right)}^{1-\left(\alpha -\beta \right)}},$$

and next

$$\underset{v\to \infty }{\lim}r_{5}\left(v,t,g\right)=\underset{v\to \infty }{lim}\frac{\tau_{r}+\left(\alpha -\beta \right)ln\left(\tau_{r}v\right)\tau_{r}}{\frac{1}{2}te^{\frac{1}{2}tv}{\left(\tau_{r}v\right)}^{1-\left(\alpha -\beta \right)}}=\underset{v\to \infty }{lim}\frac{\left(\alpha -\beta \right)}{\frac{1}{2}t\left[\frac{1}{2}tv+1-\alpha +\beta \right]{\left(\tau_{r}v\right)}^{1-\alpha +\beta }e^{\frac{1}{2}tv}}=0^{+}.$$

Applying two known inequalities $e^{-x}<\frac{k!}{k!+x^{k}}$ and [65]

(A46) $$\ln\left(x\right)\leq n\left(x^{\frac{1}{n}}-1\right),$$

being valid for any integer n,k>0 and real x>0, by putting n=1 and k=2 function $r_{5}\left(v,t,g\right)$ (A43) for any $\left(v,t,g\right)\in R_{+}\times T\times G_{1}$ can be estimated by

(A47) $$\left|r_{5}\left(v,t,g\right)\right|\leq \frac{2{\left(\tau_{r}v\right)}^{\alpha -\beta }\left|\tau_{r}v-1\right|}{2+\frac{1}{4}{\left(\frac{t}{\tau_{r}}\right)}^{2}{\left(\tau_{r}v\right)}^{2}}\leq \frac{{\left(\tau_{r}v\right)}^{\alpha -\beta }\left(\tau_{r}v+1\right)}{1+\frac{1}{8}{\left(\frac{t_{0}}{\tau_{r}}\right)}^{2}{\left(\tau_{r}v\right)}^{2}}\leq \frac{{\left(\tau_{rmax}v\right)}^{\alpha -\beta }\left(\tau_{rmax}v+1\right)}{1+\frac{1}{8}{\left(\frac{t_{0}}{\tau_{r}}\right)}^{2}{\left(\tau_{r}v\right)}^{2}}.$$

For $\tau_{rmax}v<1$, the right inequality in (A47) implies

(A48) $$\left|r_{5}\left(v,t,g\right)\right|<\frac{2}{1+\frac{1}{8}{\left(\frac{t_{0}}{\tau_{r}}\right)}^{2}{\left(\tau_{r}v\right)}^{2}}\leq 2,$$

while for $\tau_{rmax}v\geq 1$, the middle inequality in (A47) yields

(A49) $$\left|r_{5}\left(v,t,g\right)\right|\leq \frac{{\left(\tau_{r}v\right)}^{\alpha -\beta -1}+{\left(\tau_{r}v\right)}^{\alpha -\beta -2}}{\frac{1}{8}{\left(\frac{t_{0}}{\tau_{r}}\right)}^{2}}\leq \frac{2}{\frac{1}{8}{\left(\frac{t_{0}}{\tau_{r}}\right)}^{2}}\leq \frac{16}{{\left(\frac{t_{0}}{\tau_{rmax}}\right)}^{2}}=16m_{0}^{2}.$$

where positive $m_{0}$ is defined in Equation (16). Combining (A48) and (A49), we obtain the inequality

(A50) $$\left|r_{5}\left(v,t,g\right)\right|<max\left\{16m_{0}^{2},2\right\}=m_{4},$$

valid for any $\left(v,t,g\right)\in R_{+}\times T\times G_{1}$, which together with (A44) and (A45) means an absolute boundness of continuous functions $r_{12}\left(v,g\right)r_{5}\left(v,t,g\right)$ and $r_{22}\left(v,g\right)r_{5}\left(v,t,g\right)$, respectively, and in view of the absolute integrability of $v^{\alpha -1}e^{-\frac{1}{2}tv}$ and $r\left(v,g\right)v^{\alpha -1}e^{-\frac{1}{2}tv}$, imply the convergence of the integrals $I_{5}\left(t,g\right)$ (A39) and $I_{6}\left(t,g\right)$ (A40) for any t∈T and any $g\in G_{1}$. The absolute boundness of $I_{5}\left(t,g\right)$ and $I_{6}\left(t,g\right)$, uniform on the set $T\times G_{1}$, with upper bounds estimations $\frac{2\Gamma \left(\beta_{0}\right)m_{2}m_{4}}{\gamma_{1}}$ and $\frac{\pi M_{1}\left(m_{1}+m_{2}\right)m_{4}}{G_{emin}\gamma_{2}}$, respectively, follows from Property A2. Therefore, the absolute boundness of $\frac{\partial G_{M}\left(t,g\right)}{\partial \beta }$ (A32), uniform on $T\times G_{1}$**,** is proved.

#### Appendix A.1.4. Uniform on $T\times G_{1}$ Boundness of the FMM Derivative with Respect to α

The same integral properties and the spectral representation (21) will be applied to prove the boundness of the partial derivative with respect to parameter α. Differentiation (21) on both sides with respect to α yields

(A51) $$\frac{dG_{M}\left(t,g\right)}{d\alpha }=\ln\left(\tau_{r}\right)G_{M}\left(t,g\right)+G_{e}\frac{\tau_{r}^{\alpha }}{\pi }\int_{0}^{\infty }\frac{\ln\left(\tau_{r}v\right){\left(\tau_{r}v\right)}^{\alpha -\beta }\sin\left(\pi \beta \right)+\pi \cos\left(\pi \alpha \right)}{{\left(\tau_{r}v\right)}^{2\left(\alpha -\beta \right)}+2{\left(\tau_{r}v\right)}^{\alpha -\beta }\cos\left[\pi \left(\alpha -\beta \right)\right]+1}v^{\alpha -1}e^{-tv}dv-2G_{e}\frac{\tau_{r}^{\alpha }}{\pi }\int_{0}^{\infty }r\left(v,g\right)\frac{{\ln\left(\tau_{r}v\right)\left(\tau_{r}v\right)}^{2\left(\alpha -\beta \right)}+\ln\left(\tau_{r}v\right){\left(\tau_{r}v\right)}^{\alpha -\beta }\cos\left[\pi \left(\alpha -\beta \right)\right]-\pi {\left(\tau_{r}v\right)}^{\alpha -\beta }\sin\left[\pi \left(\alpha -\beta \right)\right]}{{\left(\tau_{r}v\right)}^{2\left(\alpha -\beta \right)}+2{\left(\tau_{r}v\right)}^{\alpha -\beta }\cos\left[\pi \left(\alpha -\beta \right)\right]+1}v^{\alpha -1}e^{-tv}dv+G_{e}\frac{\tau_{r}^{\alpha }}{\pi }\int_{0}^{\infty }r\left(v,g\right)v^{\alpha -1}\ln\left(v\right)e^{-tv}dv,$$

where $r\left(v,g\right)$ is described by (A12). Having in mind (A13), recalling the notations $r_{1}\left(v,g\right)$ (A14), $q\left(v,g\right)$ (A19), $r_{2}\left(v,g\right)$ (A15), $r_{4}\left(v,g\right)$ (A31), $r_{5}\left(v,t,g\right)$ (A43) and introducing the integrals

(A52) $$I_{7}\left(t,g\right)=\int_{0}^{\infty }\frac{\cos\left(\pi \alpha \right)}{q\left(v,g\right)}v^{\alpha -1}e^{-tv}dv,$$

$$I_{8}\left(t,g\right)=\int_{0}^{\infty }\;r\left(v,g\right)r_{5}\left(v,t,g\right)v^{\beta -1}e^{-\frac{1}{2}tv}dv,$$

we can express $\frac{dG_{M}\left(t,g\right)}{d\alpha }$ (A51) in a compact form as

$$\frac{dG_{M}\left(t,g\right)}{d\alpha }=G_{e}\frac{\tau_{r}^{\alpha }}{\pi }I_{5}\left(t,g\right)+G_{e}\tau_{r}^{\alpha }I_{7}\left(t,g\right)-2G_{e}\frac{\tau_{r}^{\alpha }}{\pi }I_{6}\left(t,g\right)+2G_{e}\tau_{r}^{\alpha }I_{4}\left(t,g\right)+G_{e}\frac{\tau_{r}^{\alpha }}{\pi }I_{8}\left(t,g\right),$$

where the integrals $I_{5}\left(t,g\right)$ (A35), $I_{6}\left(t,g\right)$ (A36) and $I_{4}\left(t,g\right)$ (A34) are absolutely bounded uniformly on $T\times G_{1}$. Therefore, only the convergence and boundness of the two new integrals $I_{7}\left(t,g\right)$ and $I_{8}\left(t,g\right)$ must be proved.

The boundness of $\frac{\cos\left(\pi \alpha \right)}{q\left(v,g\right)}$, uniform on $R_{+}\times G_{1}$, with the upper bound

$$\left|\frac{\cos\left(\pi \alpha \right)}{q\left(v,g\right)}\right|\leq \frac{1}{1-\cos^{2}\left(\pi \beta_{0}\right)}=m_{2},$$

combined with the absolute integrability of $v^{\alpha -1}e^{-tv}$ for any $\left(t,g\right)\in T\times G_{1}$, yields the convergence of the integral $I_{7}\left(t,g\right)$ (A52) and its absolute boundness, uniform on $T\times G_{1}$.

From (A12), the upper bound of the nonnegative function $r\left(v,g\right)$ follows

$$r\left(v,g\right)\leq \frac{1}{2-2\cos\left(\pi \beta_{0}\right)}+\frac{1}{1-\cos^{2}\left(\pi \beta_{0}\right)}=m_{1}+m_{2},$$

where constants $m_{1}$ and $m_{2}$ are defined in (A22) and (A29), respectively; therefore, the absolute boundness of $r_{5}\left(v,t,g\right)$, uniform on $R_{+}\times T\times G_{1}$ (c.f., (A50)), and integrability of $v^{\beta -1}e^{-\frac{1}{2}tv}$ imply both the convergence and the absolute boundness of $I_{8}\left(t,g\right)$, uniformly on the set $T\times G_{1}$.

The partial derivatives of the FMM with respect to the four model parameters are proved to be absolutely bounded uniformly on the set $T\times G_{1}$; therefore, the uniform boundness (22) of the gradient $\nabla_{g}G_{M}\left(t,g\right)$ follows. The theorem is proved.

### Appendix A.2. Proof of Theorem 2

Since, for any t∈T function, $G_{M}\left(t,g\right)$ (10) is differentiable with respect to g and in this case β=α, the four-element vector of model parameters g (8) is as follows

$$g={\left[\begin{array}{cccc} \alpha & \alpha & G_{e} & \tau_{r} \end{array}\right]}^{T},$$

the gradient is given by

(A53) $$\nabla_{g}G_{M}\left(t,g\right)={\left[\begin{array}{cccc} \frac{\partial G_{M}\left(t,g\right)}{\partial \alpha } & \frac{\partial G_{M}\left(t,g\right)}{\partial \alpha } & \frac{1}{2\Gamma \left(1-\alpha \right)}{\left(\frac{t}{\tau_{r}}\right)}^{-\alpha } & \frac{G_{e}\alpha }{2\Gamma \left(1-\alpha \right)t}{\left(\frac{t}{\tau_{r}}\right)}^{1-\alpha } \end{array}\right]}^{T},$$

with the partial derivative

(A54) $$\frac{\partial G_{M}\left(t,g\right)}{\partial \alpha }=\frac{G_{e}}{2\Gamma \left(1-\alpha \right)}\left[\psi \left(1-\alpha \right)+ln\left(\frac{\tau_{r}}{t}\right)\right]{\left(\frac{\tau_{r}}{t}\right)}^{\alpha },$$

where

(A55) $$\psi \left(x\right)=\frac{\Gamma^{\prime }\left(x\right)}{\Gamma \left(x\right)}$$

is the digamma (or psi) function [66] defined as the logarithmic derivative of the gamma function [66]:

$$\psi \left(x\right)=\frac{d}{dx}ln\left[\Gamma \left(x\right)\right].$$

For ${0<\beta }_{0}\leq \alpha <1$, the nonnegative digamma function $\psi \left(1-\alpha \right)$ strictly decreases from finite $\psi \left(1-\beta_{0}\right)<0$ to −∞, while the positive gamma function $\Gamma \left(1-\alpha \right)$ strictly increases from $\Gamma \left(1-\beta_{0}\right)>0$ to +∞. To evaluate the first summand of $\frac{\partial G_{M}\left(t,g\right)}{\partial \alpha }$ (A54), the following result proved by Mező and Hoffman [67] is helpful in providing an infinite product representation of the entire function $\frac{\psi \left(z\right)}{\Gamma \left(z\right)}$.

**Property** **A3** ([67] (Theorem 2.1))**.** *For all* z∈C

(A56) $$\frac{\psi \left(z\right)}{\Gamma \left(z\right)}=-e^{2\gamma z}\prod_{k=0}^{\infty }\left(1-\frac{z}{\alpha_{k}}\right)e^{z/\alpha_{k}},$$

*where* C *is the set of complex numbers,* γ *is the Euler’s constant and* $\alpha_{k}$ *are the zeros of the digamma function* $\psi \left(z\right)$.

It is known [67] that the zeros $\alpha_{k}$ are real, and all but one are negative; here, $\alpha_{0}$ is the positive zero, and $\alpha_{1}$,$\;\alpha_{2},\ldots$ are the negative ones in decreasing order. The course of $\frac{\psi \left(z\right)}{\Gamma \left(z\right)}$ for the real 0≤z≤1 is illustrated by Figure A1. For α=1, by (A56), the quotient $\frac{\psi \left(1-\alpha \right)}{\Gamma \left(1-\alpha \right)}$ is as follows $\frac{\psi \left(0\right)}{\Gamma \left(0\right)}=-1$, while for α=β→0, the quotient $\frac{\psi \left(1-\alpha \right)}{\Gamma \left(1-\alpha \right)}$ tends to $\frac{\psi \left(1\right)}{\Gamma \left(1\right)}=-\gamma$. Therefore, for any $\left(t,g\right)\in T\times G_{2}$

$$\left|\frac{E\psi \left(1-\alpha \right)}{2\Gamma \left(1-\alpha \right)}{\left(\frac{\tau_{r}}{t}\right)}^{\alpha }\right|\leq \frac{G_{emax}}{2}\frac{\tau_{rmax}}{t_{0}},$$

i.e., the first summand of $\frac{\partial G_{M}\left(t,g\right)}{\partial \alpha }$ (A54) is bounded.

Using the inequality (A46), the second summand of $\frac{\partial G_{M}\left(t,g\right)}{\partial \alpha }$ (A54) can be estimated as follows

$$\frac{G_{e}}{2\Gamma \left(1-\alpha \right)}ln\left(\frac{\tau_{r}}{t}\right){\left(\frac{\tau_{r}}{t}\right)}^{\alpha }\leq \frac{mG_{e}}{2\Gamma \left(1-\alpha \right)}\left[{\left(\frac{\tau_{r}}{t}\right)}^{\frac{1}{m}}-1\right]{\left(\frac{\tau_{r}}{t}\right)}^{\alpha },$$

where an integer m>0. From the above, for $\left(t,g\right)\in T\times G_{2}$, the next inequality follows

$$\frac{G_{e}}{2\Gamma \left(1-\alpha \right)}ln\left(\frac{\tau_{r}}{t}\right){\left(\frac{\tau_{r}}{t}\right)}^{\alpha }\leq \frac{m\;G_{emax}}{2}\left|{\left(\frac{\tau_{rmax}}{t_{0}}\right)}^{\frac{1}{m}}-1\right|\frac{\tau_{rmax}}{t_{0}},$$

i.e., the second summand of $\frac{\partial G_{M}\left(t,g\right)}{\partial \alpha }$ (A54) is bounded, too.

Since, for $0<\beta_{0}\leq \;\alpha \leq 1$ gamma function $\Gamma \left(1-\alpha \right)\geq \Gamma \left(1-\beta_{0}\right)>1$, the last two elements of the gradient (A53) are nonnegative definite (c.f., Property 1) and bounded by $\frac{1}{2}\frac{\tau_{rmax}}{t_{0}}$ and $\frac{G_{emax}}{2t_{0}}\frac{\tau_{rmax}}{t_{0}}$, respectively, for any $\left(t,g\right)\in T\times G_{2}$. The theorem is proved.

## Appendix B

### Appendix B.1. The Results of the Numerical Studies for Material I

**Table A1:** The elements $\alpha_{N}^{*}$, $\beta_{N}^{*}$, $G_{eN}^{*}$ and $\tau_{rN}^{*}$ of the FMM parameter vector $g_{N}^{*}$ solving identification task (25) for real relaxation modulus (36) of the material described by the unimodal Gauss-like distribution, the mean-square identification indices $Q_{N}\left(g_{N}^{*}\right)$, Equation (24), the mean relative square model approximation index $Q_{Nrel}\left(g_{N}^{*}\right)$, Equation (38), the sampling points-independent integral indices $Q\left(g_{N}^{*}\right)$ defined by the optimization task (26), and the relative errors ERR (37) of the FMM parameter $g^{*}$ approximation for N relaxation modulus measurements independently disturbed by additive, zero mean, normally distributed noises with standard deviation $\sigma =2\;\left[kPa\right]$.

N	$Q_{N}\left(g_{N}^{*}\right)\;\left[{kPa}^{2}\right]$	$Q_{Nrel}\left(g_{N}^{*}\right)\left[-\right]$	$Q\left(g_{N}^{*}\right)\;\left[{kPa}^{2}\right]$	ERR [%]	$\alpha_{N}^{*}\left[-\right]$	$\beta_{N}^{*}\left[-\right]$	$G_{eN}^{*}\;\left[kPa\right]$	$\tau_{rN}^{*}\left[s\right]$
50	9.295186 × 10^−5^	2.102093 × 10^−3^	2.863765 × 10^−3^	0.527	0.963788	8.644275 × 10^−2^	2.862573	15.170858
100	5.614324 × 10^−4^	5.907274 × 10^−5^	1.350707 × 10^−3^	1.224	0.965614	7.207174 × 10^−2^	2.745219	16.225154
200	2.185904 × 10^−4^	1.109724 × 10^−4^	4.959376 × 10^−3^	4.537 × 10^−3^	0.954725	8.815551 × 10^−2^	3.065976	14.289093
500	5.559271 × 10^−4^	5.053629 × 10^−5^	5.262647 × 10^−4^	7.740 × 10^−3^	0.915232	1.261495 × 10^−2^	3.113879	12.68090
1000	4.594139 × 10^−4^	1.476206 × 10^−4^	5.268387 × 10^−4^	3.104 × 10^−4^	0.920937	1.341017 × 10^−2^	3.081285	12.926718
2000	4.668059 × 10^−4^	3.243456 × 10^−6^	5.396114 × 10^−4^	5.315 × 10^−2^	0.929099	2.375304 × 10^−2^	3.015563	13.557098
5000	5.289533 × 10^−4^	3.282759 × 10^−7^	5.213213 × 10^−4^	2.876 × 10^−4^	0.920502	1.445854 × 10^−2^	3.081489	12.984136
7000	5.224292 × 10^−4^	3.978418 × 10^−6^	5.215398 × 10^−4^	1.654 × 10^−4^	0.920364	1.419721 × 10^−2^	3.082754	12.979817
10,000	5.082251 × 10^−4^	3.739909 × 10^−6^	5.219846 × 10^−4^	9.301 × 10^−5^	0.920327	1.391317 × 10^−2^	3.083746	12.942422
12,000	5.150186 × 10^−4^	1.363350 × 10^−5^	5.208965 × 10^−4^	1.689 × 10^−5^	0.920187	1.504288 × 10^−2^	3.085455	12.942422
15,000	5.247854 × 10^−4^	3.687418 × 10^−6^	5.205439 × 10^−4^	1.938 × 10^−8^	0.920014	1.472034 × 10^−2^	3.086680	12.949456

**Table A2:** The elements $\alpha_{N}^{*}$, $\beta_{N}^{*}$, $G_{eN}^{*}$ and $\tau_{rN}^{*}$ of the FMM parameter vector $g_{N}^{*}$ solving identification task (25) for real relaxation modulus (36) of the material described by the unimodal Gauss-like distribution, the mean-square identification indices $Q_{N}\left(g_{N}^{*}\right)$, Equation (24), the mean relative square model approximation index $Q_{Nrel}\left(g_{N}^{*}\right)$, Equation (38), the sampling points-independent integral indices $Q\left(g_{N}^{*}\right)$ defined by the optimization task (26), and the relative errors ERR (37) of the FMM parameter $g^{*}$ approximation for N measurements independently disturbed by additive, zero mean, normally distributed noises with standard deviation $\sigma =5\;\left[kPa\right]$.

N	$Q_{N}\left(g_{N}^{*}\right)\;\left[k{Pa}^{2}\right]$	$Q_{Nrel}\left(g_{N}^{*}\right)\left[-\right]$	$Q\left(g_{N}^{*}\right)\;\left[{kPa}^{2}\right]$	ERR [%]	$\alpha_{N}^{*}\left[-\right]$	$\beta_{N}^{*}\left[-\right]$	$G_{eN}^{*}\;\left[kPa\right]$	$\tau_{rN}^{*}\left[s\right]$
50	1.246174 × 10^−4^	3.1182072 × 10^−3^	2.953417 × 10^−3^	0.501	0.962059	8.720788 × 10^−2^	2.868287	15.170858
100	6.042548 × 10^−4^	9.788569 × 10^−3^	9.505292 × 10^−4^	0.542	0.950711	5.528005 × 10^−2^	2.859441	15.193338
200	2.469407 × 10^−4^	2.622478 × 10^−4^	4.888504 × 10^−3^	3.999 × 10^−3^	0.954563	8.747687 × 10^−2^	3.067250	14.289093
500	5.754599 × 10^−4^	1.136117 × 10^−7^	5.288353 × 10^−4^	1.1543 × 10^−4^	0.917799	1.594113 × 10^−2^	3.090037	12.837417
1000	4.8724914 × 10^−4^	6.769619 × 10^−4^	5.261736 × 10^−4^	2.129 × 10^−4^	0.921142	1.374989 × 10^−2^	3.082219	12.926718
2000	4.8687842 × 10^−4^	3.040833 × 10^−7^	5.400918 × 10^−4^	5.345 × 10^−2^	0.929234	2.394509 × 10^−2^	3.015365	13.557098
5000	5.4973567 × 10^−4^	3.940686 × 10^−6^	5.211862 × 10^−4^	3.026 × 10^−4^	0.920472	1.459525 × 10^−2^	3.081354	12.984135
7000	5.401956 × 10^−4^	1.559930 × 10^−6^	5.214093 × 10^−4^	1.573 × 10^−4^	0.920350	1.428889 × 10^−2^	3.082852	12.979817
10,000	5.261243 × 10^−4^	2.744246 × 10^−6^	5.221499 × 10^−4^	5.516 × 10^−4^	0.920905	1.446157 × 10^−2^	3.079473	12.979817
12,000	5.356062 × 10^−4^	5.782985 × 10^−5^	5.208668 × 10^−4^	2.386 × 10^−5^	0.920122	1.495904 × 10^−2^	3.085216	12.942422
15,000	5.457918 × 10^−4^	4.535517 × 10^−6^	5.205517 × 10^−4^	2.513 × 10^−7^	0.919980	1.477095 × 10^−2^	3.086569	12.949456

**Table A3:** The elements $\alpha_{N}^{*}$, $\beta_{N}^{*}$, $G_{eN}^{*}$ and $\tau_{rN}^{*}$ of the FMM parameter vector $g_{N}^{*}$ solving identification task (25) for real relaxation modulus (36) of the material described by the unimodal Gauss-like distribution, the mean relative square model approximation index $Q_{Nrel}\left(g_{N}^{*}\right)$, Equation (38), the sampling points-independent integral indices $Q\left(g_{N}^{*}\right)$ defined by the optimization task (26), and the relative errors ERR (37) of the parameter $g^{*}$ approximation for N measurements independently disturbed by additive, zero mean, normally distributed noises with standard deviation $\sigma =8\;\left[kPa\right]$.

N	$Q_{N}\left(g_{N}^{*}\right)\;\left[{kPa}^{2}\right]$	$Q_{Nrel}\left(g_{N}^{*}\right)\left[-\right]$	$Q\left(g_{N}^{*}\right)\;\left[k{Pa}^{2}\right]$	ERR [%]	$\alpha_{N}^{*}\left[-\right]$	$\beta_{N}^{*}\left[-\right]$	$G_{eN}^{*}\;\left[kPa\right]$	$\tau_{rN}^{*}\left[s\right]$
50	1.823164 × 10^−4^	4.314509 × 10^−3^	2.993805 × 10^−3^	0.504	0.961565	8.769949 × 10^−2^	2.867641	15.1708579
100	6.647523 × 10^−4^	0.353591	9.411604 × 10^−4^	0.517	0.949857	5.428199 × 10^−2^	2.864822	15.193338
200	2.961081 × 10^−4^	4.334879 × 10^−4^	4.686224 × 10^−3^	4.277 × 10^−3^	0.954203	8.597868 × 10^−2^	3.066582	14.289093
500	6.078210 × 10^−4^	2.946700 × 10^−5^	5.271033 × 10^−4^	3.193 × 10^−4^	0.918084	1.579814 × 10^−2^	3.092238	12.837417
1000	5.328032 × 10^−4^	2.789884 × 10^−3^	5.267889 × 10^−4^	1.300 × 10^−4^	0.921325	1.356372 × 10^−2^	3.083203	12.926718
2000	5.248407 × 10^−4^	3.026647 × 10^−7^	5.403471 × 10^−4^	5.202 × 10^−2^	0.929297	2.403479 × 10^−2^	3.016320	13.557098
5000	5.883023 × 10^−4^	1.431097 × 10^−5^	5.210845 × 10^−4^	3.179 × 10^−4^	0.920442	1.473303 × 10^−2^	3.081219	12.984135
7000	5.759879 × 10^−4^	1.780341 × 10^−7^	5.220610 × 10^−4^	2.087 × 10^−3^	0.921658	1.538542 × 10^−2^	3.072622	13.066791
10,000	5.616302 × 10^−4^	2.127802 × 10^−6^	5.220589 × 10^−4^	5.578 × 10^−4^	0.920855	1.448935 × 10^−2^	3.079433	12.979817
12,000	5.739756 × 10^−4^	2.163690 × 10^−4^	5.211071 × 10^−4^	8.574 × 10^−4^	0.921109	1.594595 × 10^−2^	3.077685	13.006321
15,000	5.843841 × 10^−4^	5.116492 × 10^−6^	5.207537 × 10^−4^	4.857 × 10^−4^	0.920823	1.552819 × 10^−2^	3.079921	13.006321

### Appendix B.2. The Results of the Numerical Studies for Material II

**Table A4:** For the optimal FMM approximating the relaxation modulus (43) of the material described by the BSW spectrum (42): the elements $\alpha_{N}^{*}$ $\beta_{N}^{*}$, $G_{eN}^{*}$ and $\tau_{rN}^{*}$ of the vector $g_{N}^{*}$ solving identification task (25), the mean-square identification indices $Q_{N}\left(g_{N}^{*}\right)$, Equation (24), the mean relative square model approximation index $Q_{Nrel}\left(g_{N}^{*}\right)$, Equation (38), the sampling points-independent integral indices $Q\left(g_{N}^{*}\right)$ defined by the optimization task (26), and the relative errors ERR (37) of the parameter $g^{*}$ approximation for N relaxation modulus measurements independently disturbed by additive normally distributed noises with standard deviation $\sigma =3\;\left[kPa\right]$.

N	$Q_{N}\left(g_{N}^{*}\right)\;\left[{MPa}^{2}\right]$	$Q_{Nrel}\left(g_{N}^{*}\right)\left[-\right]$	$Q\left(g_{N}^{*}\right)\;\left[{MPa}^{2}\right]$	ERR [%]	$\alpha_{N}^{*}\left[-\right]$	$\beta_{N}^{*}\left[-\right]$	$G_{eN}^{*}\;\left[MPa\right]$	$\tau_{rN}^{*}\left[s\right]$
50	1.315712 × 10^−5^	3.597318 × 10^−7^	5.638335 × 10^−5^	2.342	0.677682	6.870627 × 10^−2^	1.370585	5.418555 × 10^3^
100	1.161929 × 10^−5^	5.298049 × 10^−7^	4.939762 × 10^−5^	4.151	0.656134	6.493121 × 10^−2^	1.409899	5.094185 × 10^3^
200	1.03161 × 10^−5^	1.124491 × 10^−8^	5.287861 × 10^−5^	4.759	0.65004	6.373051 × 10^−2^	1.421409	5.002011 × 10^3^
500	9.772230 × 10^−6^	2.140316 × 10^−8^	2.966257 × 10^−5^	0.897	0.686475	7.329572 × 10^−2^	1.336261	5.791700 × 10^3^
1000	1.216504 × 10^−5^	1.472521 × 10^−8^	2.964808 × 10^−5^	0.847	0.691723	7.332173 × 10^−2^	1.331643	5.808836 × 10^3^
2000	9.462435 × 10^−6^	2.397636 × 10^−9^	3.703687 × 10^−5^	1.928	0.674709	6.955942 × 10^−2^	1.364964	5.509183 × 10^3^
5000	3.372717 × 10^−5^	1.236704 × 10^−8^	2.439945 × 10^−5^	0.061	0.750336	8.307524 × 10^−2^	1.245023	6.555588 × 10^3^
7000	3.499392 × 10^−5^	3.0361484 × 10^−9^	2.578132 × 10^−5^	0.179	0.761153	8.483605 × 10^−2^	1.231209	6.669047 × 10^3^
10,000	3.974638 × 10^−5^	1.327619 × 10^−9^	2.524376 × 10^−5^	0.136	0.757999	8.429268 × 10^−2^	1.235341	6.633616 × 10^3^
12,000	3.289041 × 10^−5^	2.540537 × 10^−10^	2.384835 × 10^−5^	1.384 × 10^−3^	0.735839	8.062010 × 10^−2^	1.265447	6.373837 × 10^3^
15,000	3.259757 × 10^−5^	2.789747 × 10^−10^	2.383509 × 10^−5^	6.536 × 10^−5^	0.737489	8.098389 × 10^−2^	1.262561	6.402808 × 10^3^

**Table A5:** For the optimal FMM approximating the relaxation modulus (43) of the material described by the BSW spectrum (42): the elements $\alpha_{N}^{*}$, $\beta_{N}^{*}$, $G_{eN}^{*}$ and $\tau_{rN}^{*}$ of the parameter vector $g_{N}^{*}$, the mean-square identification indices $Q_{N}\left(g_{N}^{*}\right)$, Equation (24), the mean relative square model approximation index $Q_{Nrel}\left(g_{N}^{*}\right)$, Equation (38), the sampling points-independent integral indices $Q\left(g_{N}^{*}\right)$ defined by the optimization task (26), and the relative errors ERR (37) of the parameter $g^{*}$ for N measurements corrupted by the noises with standard deviation $\sigma =6\;\left[kPa\right]$.

N	$Q_{N}\left(g_{N}^{*}\right)\;\left[{MPa}^{2}\right]$	$Q_{Nrel}\left(g_{N}^{*}\right)\left[-\right]$	$Q\left(g_{N}^{*}\right)\;\left[{MPa}^{2}\right]$	ERR [%]	$\alpha_{N}^{*}\left[-\right]$	$\beta_{N}^{*}\left[-\right]$	$G_{eN}^{*}\;\left[MPa\right]$	$\tau_{rN}^{*}\left[s\right]$
50	5.334837 × 10^−5^	1.607199 × 10^−6^	5.383509 × 10^−5^	1.783	0.691583	7.062629 × 10^−2^	1.352647	5.543339 × 10^3^
100	4.625811 × 10^−5^	1.912785 × 10^−6^	5.226179 × 10^−5^	4.853	0.652363	6.407151 × 10^−2^	1.421237	4.988252 × 10^3^
200	4.104462 × 10^−5^	6.4067158 × 10^−8^	6.964808 × 10^−5^	7.841	0.632293	5.905374 × 10^−2^	1.467987	4.606199 × 10^3^
500	3.664178 × 10^−5^	7.819189 × 10^−8^	3.074233 × 10^−5^	1.039	0.681071	7.285674 × 10^−2^	1.342891	5.745449 × 10^3^
1000	3.879589 × 10^−5^	6.729167 × 10^−8^	2.961505 × 10^−5^	0.869	0.689812	7.328929 × 10^−2^	1.333233	5.801105 × 10^3^
2000	3.618881 × 10^−5^	1.385717 × 10^−9^	3.604767 × 10^−5^	1.786	0.675299	6.992477 × 10^−2^	1.362099	5.542684 × 10^3^
5000	5.999448 × 10^−5^	3.264396 × 10^−8^	2.456599 × 10^−5^	8.109 × 10^−2^	0.752093	8.335898 × 10^−2^	1.242558	6.579817 × 10^3^
7000	6.106792	5.065819 × 10^−9^	2.598612 × 10^−5^	0.195	0.763258	8.506666 × 10^−2^	1.229229	6.680137 × 10^3^
10,000	6.592012 × 10^−5^	1.717204 × 10^−9^	2.536338 × 10^−5^	0.144	0.759401	8.444666 × 10^−2^	1.234044	6.640191 × 10^3^
12,000	5.916358 × 10^−5^	2.831521 × 10^−9^	2.384641 × 10^−5^	5.855 × 10^−4^	0.737150	8.077941 × 10^−2^	1.264056	6.382156 × 10^3^
15,000	5.893401 × 10^−5^	1.984301 × 10^−10^	2.384002 × 10^−5^	2.644 × 10^−4^	0.738283	8.108685 × 10^−2^	1.261701	6.408038 × 10^3^

**Table A6:** For the optimal FMM approximating the relaxation modulus (43) of the material described by the BSW spectrum (42): the elements $\alpha_{N}^{*}$, $\beta_{N}^{*}$, $G_{eN}^{*}$ and $\tau_{rN}^{*}$ of the parameter vector $g_{N}^{*}$, the mean-square identification indices $Q_{N}\left(g_{N}^{*}\right)$, Equation (24), the mean relative square model approximation index $Q_{Nrel}\left(g_{N}^{*}\right)$, Equation (38), the sampling points-independent integral indices $Q\left(g_{N}^{*}\right)$ defined by the optimization task (26), and the relative errors ERR (37) of the parameter $g^{*}$ for N measurements corrupted by the noises with standard deviation $\sigma =8\;\left[kPa\right]$.

N	$Q_{N}\left(g_{N}^{*}\right)\;\left[{MPa}^{2}\right]$	$Q_{Nrel}\left(g_{N}^{*}\right)\left[-\right]$	$Q\left(g_{N}^{*}\right)\;\left[M{Pa}^{2}\right]$	ERR [%]	$\alpha_{N}^{*}\left[-\right]$	$\beta_{N}^{*}\left[-\right]$	$G_{eN}^{*}\;\left[MPa\right]$	$\tau_{rN}^{*}\left[s\right]$
50	9.520744 × 10^−5^	2.941612 × 10^−6^	6.384002 × 10^−5^	1.448	0.701586	7.198997 × 10^−2^	1.340251	5.627729 × 10^3^
100	8.218302 × 10^−5^	3.329357 × 10^−6^	5.485285 × 10^−5^	5.348	0.649888	6.349515 × 10^−2^	1.428867	4.918087 × 10^3^
200	7.290485 × 10^−5^	1.233057 × 10^−7^	8.330015 × 10^−5^	10.352	0.620745	5.578770 × 10^−2^	1.501144	4.339259 × 10^3^
500	6.446603 × 10^−5^	1.355064 × 10^−7^	3.166061 × 10^−5^	1.145	0.677484	7.255754 × 10^−2^	1.347428	5.713136 × 10^3^
1000	6.646278 × 10^−5^	1.240244 × 10^−7^	2.966288 × 10^−5^	0.885	0.688528	7.326539 × 10^−2^	1.334319	5.795691 × 10^3^
2000	6.395292 × 10^−5^	2.673029 × 10^−9^	3.542823 × 10^−5^	1.693	0.675699	7.016833 × 10^−2^	1.360185	5.565162 × 10^3^
5000	8.738813 × 10^−5^	5.134265 × 10^−8^	2.469399 × 10^−5^	9.607 × 10^−2^	0.753278	8.354811 × 10^−2^	1.240915	6.595934 × 10^3^
7000	8.835062 × 10^−5^	6.705427 × 10^−9^	2.613160 × 10^−5^	0.205	0.764677	8.522041 × 10^−2^	1.227913	6.687353 × 10^3^
10,000	9.319389 × 10^−5^	2.003354 × 10^−9^	2.544649 × 10^−5^	0.149	0.760331	8.454841 × 10^−2^	1.233188	6.644512 × 10^3^
12,000	8.640779 × 10^−5^	6.119895 × 10^−9^	2.384921 × 10^−5^	2.494 × 10^−4^	0.738014	8.088431 × 10^−2^	1.263145	6.387532 × 10^3^
15,000	8.625405 × 10^−5^	1.518245 × 10^−10^	2.384484 × 10^−5^	4.544 × 10^−4^	0.738792	8.115159 × 10^−2^	1.261157	6.411273 × 10^3^
